# Spatio-genetically coordinated TPR domain-containing proteins modulate c-di-GMP signaling in *Vibrio vulnificus*

**DOI:** 10.1371/journal.ppat.1013353

**Published:** 2025-07-16

**Authors:** Shobnom Mustaree, Ram Podicheti, Doug Rusch, Dean A. Rowe-Magnus

**Affiliations:** 1 Department of Biology, Indiana University Bloomington, Bloomington, Indiana, United States of America; 2 Center for Genomics and Bioinformatics, Indiana University Bloomington, Bloomington, Indiana, United States of America; University of Pennsylvania Perelman School of Medicine, UNITED STATES OF AMERICA

## Abstract

*Vibrio* species, which include several pathogens, are autochthonous to estuarine and warm coastal marine environments, where biofilm formation bolsters their ecological persistence and transmission. Here, we identify a bicistronic operon, *rcbAB*, whose products synergistically inhibit motility and promote biofilm maturation post-attachment by modulating intracellular c-di-GMP levels in the human and animal pathogen *V. vulnificus*. RcbA contains an N-terminal tetratricopeptide repeat (TPR) domain and a structured C-terminal region of unknown function, while RcbB possesses an N-terminal TPR domain and a C-terminal GGDEF domain characteristic of diguanylate cyclases. The TPR domain of RcbB represses its diguanylate cyclase activity, while RcbA’s TPR domain and C-terminal region co-operatively de-repress it. Localization of both proteins to the flagellar pole is TPR-dependent but not co-dependent, although RcbA anchors RcbB to the pole in the absence of polar landmarks such as HubP and flagella. The conservation of *rcbAB* across diverse bacterial taxa substantiates its fundamental importance in bacterial biology. This work demonstrates how spatio-genetically coordinated TPR domain-containing proteins modulate c-di-GMP signaling, contributing to our understanding of biofilm formation in *Vibrio* species and potentially other bacteria. It also reveals the first evidence of inter-protein interaction via the TPR domains of both partners, challenging the conventional paradigm in which only one bears the domain.

## Author summary

This study identifies the *rcbAB* operon as a critical regulator of biofilm development in the human and animal pathogen, *V. vulnificus*. The operon encodes tetratricopeptide repeat (TPR) domain-containing proteins that exhibit novel complex interactions and regulatory mechanisms. The conservation of this operon across diverse bacterial taxa supports its fundamental importance in bacterial biology. These findings expand our understanding of TPR domain versatility in mediating inter-protein interactions and provide new insights into complex formation and cellular signaling pathways in bacteria, which has potential implications for our comprehension of bacterial biofilm formation and may inform future strategies for controlling bacterial infections.

## Introduction

*Vibrios* are Gram-negative, motile, halophilic, facultatively anaerobic bacteria that inhabit estuaries and warm coastal waters [1]. A dozen *Vibrio* species are pathogenic [2] and their environmental persistence and transmission are bolstered by their ability to form biofilms on biotic and abiotic marine surfaces [3,4]. Infection typically occurs through the consumption of contaminated seafood or exposure to contaminated water [5,6]. As seafood is a widely consumed and economically important food source, contamination by *Vibrio* species poses a serious public health risk. Among the pathogenic species, *V. cholerae*, *V. parahaemolyticus* and *V. vulnificus* are the primary contributors to the rising global prevalence of seafood-borne infections [7]. *V. cholerae*, the causative agent of cholera, causes a severe diarrheal illness that can result in fatal outcomes if left untreated [8]. *V. parahaemolyticus* is predominantly linked to acute gastroenteritis [7] and *V. vulnificus* causes life-threatening septicemia and wound infections [9,10]. It is an opportunistic pathogen that is responsible for over 95% of seafood-related deaths in the Unites States [11]. *V. vulnificus* carries the highest death rate and per case economic burden (>$3.3 M) of any foodborne disease agent, with an estimated annual cost of US $320 million [11–13]. It colonizes plankton, shrimp, fish, and eels [14–20] and forms biofilms in bivalves such as oysters that concentrate it from the surrounding waters when filter-feeding [17,21,22].

A biofilm is a complex community of microorganisms where normally free-swimming bacteria exist as part of a sessile, surface-associated community [23] that increases access to nutritional resources, provides protection from environmental insults and predation, and underscores environmental persistence [24], symbiosis [25], lateral gene transfer [26], and the establishment of chronic infections [27]. Biofilm formation is tightly controlled at multiple stages, from initial surface approach and attachment to irreversible adhesion and maturation, culminating in biofilm dispersal. Each stage involves precise molecular signaling and regulatory mechanisms that govern bacterial behavior, colonization, and community development within the biofilm structure [28].

The secondary signaling molecule bis-(3’-5’)-cyclic dimeric guanosine monophosphate (c-di-GMP) is a key regulator of biofilm formation in many bacteria [29]. It is synthesized by diguanylate cyclases (DGCs) bearing a GGDEF domain and degraded by phosphodiesterases (PDEs) containing EAL or HD-GYP domains [30,31]. Increases in c-di-GMP concentration generally repress flagellar-mediated swimming motility and virulence gene expression, while enhancing extracellular polysaccharide (EPS) production and biofilm formation [32–34]. DGC and PDE domains are often found in combination with other domains (e.g., CACHE, PAS, GAF, PBP, CSS, and GCS domains that sense amino acids, nucleotides, light, redox, and oxygen) that enable integration of multiple environmental signals and regulatory inputs to facilitate precise control of c-di-GMP levels in the cell [35,36]. A variety of effectors that bind c-di-GMP and elicit a physiological response have been identified [37]. These include proteins containing degenerate GGDEF and EAL domains [38], the PilZ domain [39], the GIL domain [40], motifs (e.g., I-site, Walker A and W[F/L/M][T/S]R [41–46]) and riboswitches [47–49]. These binding sites can be found alone [50,51] or coupled to output domains, the function of which is regulated by signal binding [52–55].

In *V. vulnificus*, environmental signals such as changes in calcium, sulfate and temperature can induce biofilm formation by increasing cellular c-di-GMP [56–60]. In response, the regulator BrpR activates expression of *brpT*, and together these regulators induce the production of BRP EPS [61–65] and calcium-binding matrix proteins that promote biofilm formation [57,58,63,66]. c-di-GMP is also bound by PlzD, a YcgR homolog [67,68] that localizes to the flagellar pole to slows swimming speed and turn frequency of motile cells [69].

Tetratricopeptide repeats (TPRs) represent a structural motif initially identified in yeast [70] that have since been found across a diverse array of proteins in all three domains of life [71], and represent an elegant protein-protein interaction module for the assembly and localization of multiprotein complexes within cells [72,73]. These motifs are characterized by a loosely conserved sequence of 34 amino acids, featuring alternating large and small side-chain residues [72,74]. Despite considerable sequence variability among TPRs, structural analyses reveal a highly conserved three-dimensional conformation [75,76]. Each TPR motif comprises two anti-parallel α-helices connected by a turn. Typically, TPR motifs are arranged in tandem repeats of three to sixteen units aligned at regular angles to form a right-handed superhelix, resulting in a binding groove that provides an extensive surface area for interaction with ligands and other proteins. TPR domain-containing proteins often play vital roles in eukaryotic cell processes, such as gene regulation, steroid receptor function, and protein import [73,77,78]. The importance of TPR-containing proteins is highlighted by the discovery that mutations in the TPR region are linked to several severe human diseases ranging from retinopathy to Down syndrome [79–81]. Strikingly, in most cases the target protein of eukaryotic TPR proteins is unknown. In bacteria, TPR domain-containing proteins have been reported to be directly involved in virulence-associated functions, including the translocation of virulence factors into host cells, adhesion to host cells, and blocking of phagolysosomal maturation [82–86].

Here, we screened a *V. vulnificus* transposon library for mutants deficient in calcium-induced biofilm formation and identified a two-gene operon, herein designated *rcbA* and *rcbB* (regulator of calcium-dependent biofilm development), that regulates biofilm formation and motility behavior. RcbA bears a putative N-terminal TPR domain and structured C-terminal region of unknown function, and RcbB harbors a N-terminal TPR domain and a C-terminal GGDEF domain found in DGCs. We show that RcbA and RcbB co-operatively increase c-di-GMP levels to enhance biofilm maturation post-attachment and slow motility. We demonstrate that the TPR domain of RcbB represses its DGC activity, while RcbA de-represses it. The TPR domains of RcbA and RcbB are crucial for their function, localizing both proteins to the flagellar pole and mediating their interaction. Polar localization of RcbA and RcbB is not co-dependent, but notably, RcbA anchors RcbB to the pole in the absence of polar landmarks such as HubP and flagella. To the best of our knowledge, this observation represents a novel instance of protein-protein interaction mediated by the tetratricopeptide repeat (TPR) domains of both protein partners, contrasting with the conventional paradigm in which only one interacting partner bears a TPR domain. The discovery of this mutual TPR-TPR interaction mechanism expands our knowledge of the versatility of TPR domains in facilitating protein-protein interactions and may have implications for our understanding of protein complex formation and cellular signaling pathways. Finally, the conservation and synteny of the *rcbAB* operon across diverse bacterial taxa implies that the functions of RcbA and RcbB are fundamentally important for the organisms in which they reside.

## Results

### A bicistronic operon mediates calcium-induced biofilm development in *V. vulnificus*

**Fig 1:**
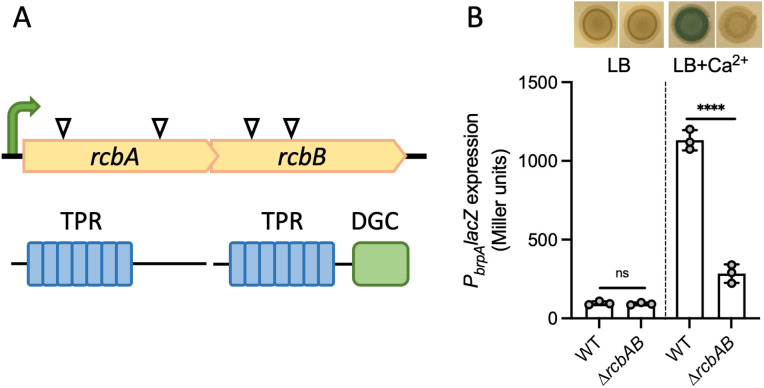
The *rcbAB* operon regulates calcium-induced *brp* exopolysaccharide expression. A, genetic organization of *rcbAB.* White arrows indicate Tn insertions identified in independent mutants and the bent green arrow represents a putative promoter. The domain structure of the encoded proteins is below. A tetratricopeptide (TPR) domain containing 7 repeats (blue) is present in both proteins, along with a diguanylate cyclase (DGC) domain (green) in RcbB. B, representative images of growth of wildtype *V. vulnificus* (WT) and ∆*rcbAB* mutant (Tn) strains bearing a *P*_*brpA*_*lacZ* reporter on LB without or supplemented with 15 mM CaCl_2_ (LB + Ca^2+^). A plot of the corresponding *P*_*brpA*_*lacZ* expression levels is below. Bars indicate the respective mean values and error bars represent the standard deviation of triplicate assays. Statistically significant differences between samples (****p < 0.0001; ns, no significant difference) were determined by unpaired Student’s t-test (two-tailed).

A *V. vulnificus* transposon (Tn) library was screened to identify mutants deficient in calcium-induced biofilm formation. Whole genome sequencing revealed multiple Tn insertions in an operon comprising two genes, *aot11_00230* and *aot11_00235*, herein designated *rcbA* and *rcbB* (Fig 1A). In silico analysis predicted RcbA and RcbB to be cytoplasmic, with RcbA containing an N-terminal TPR domain and C-terminal structured region of unknown function, and RcbB possessing both an N-terminal TPR domain and a C-terminal DGC domain. To ascertain the impact of *rcbAB* on the production of biofilm matrix components, wildtype and Δ*rcbAB* strains harboring a *P*_*brpA*_*lacZ* reporter fusion (*P*_*brpA*_ is a c-di-GMP responsive promoter that drives the expression of genes responsible for synthesizing BRP, a biofilm-promoting EPS) were cultured in LB media with or without calcium supplementation. Whilst *P*_*brpA*_ expression was induced >12-fold in wildtype cells by the addition of calcium, deletion of *rcbAB* suppressed this phenotype (Fig 1B). A similar expression pattern was observed for *P*_*cabA*_, a c-di-GMP responsive promoter that drives expression of the calcium-binding matrix protein, CabA (S1 Fig). Collectively, these findings suggested that *rcbAB* encodes regulators of BRP and CabA production, critical components of *V. vulnificus* biofilms.

**Fig 2:**
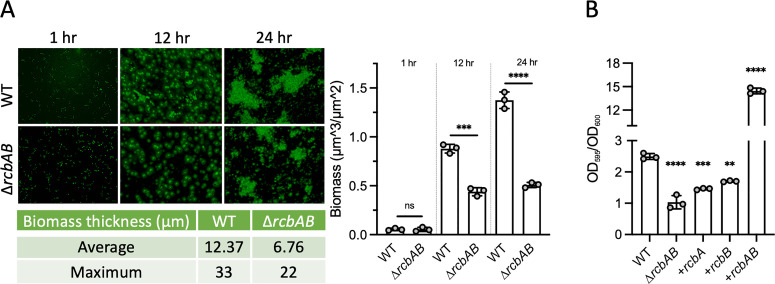
RcbA and RcbB co-operatively promote biofilm development post-attachment. A, representative fluorescence images of biofilms formed by wildtype (WT) and ∆*rcbAB* strains under constant flow in IO20YP after 1, 12 and 24 hours. The average and maximum biomass thickness is shown below and a plot of the biomass for the same strains is on the right. B, biofilm formation (OD_595_/OD_600_) by WT and ∆*rcbAB* strains expressing (+) *rcbA*, *rcbB* or both genes. Bars indicate the respective mean values and error bars represent the standard deviation of triplicate assays. Statistically significant differences were determined by an unpaired Student’s t-test (two-tailed) in A and relative to WT cells by one-way ANOVA with a Tukey’s multiple comparisons post-hoc test in B (**p < 0.01, ***p < 0.001, ****p < 0.0001; ns, no significant difference). Expression was induced with 1 µM IPTG.

To determine the stage at which *rcbAB* impacted biofilm formation, initial surface attachment and subsequent biofilm development of wildtype and ∆*rcbAB* strains were monitored under continuous flow conditions in an estuarine medium, IO20YP. After 1 hour of incubation, both wildtype and ∆*rcbAB* strains exhibited comparable numbers of single cells attached to the surface (Fig 2A). However, at 12- and 24-hours post-attachment, the biomass of the ∆*rcbAB* strain was significantly reduced, measuring 2-fold and 2.7-fold lower than that of the wildtype strain, respectively. Furthermore, the average and maximum biomass thickness of ∆*rcbAB* biofilms were 2.7-fold and 3.2-fold lower, respectively, than those observed in wildtype biofilms. Expression of either *rcbA* or *rcbB* alone in the ∆*rcbAB* background did not restore biofilm formation to wildtype levels; however, their co-expression resulted in a 5-fold increase in biofilm formation (Fig 2B), indicating a potential co-operative interaction between RcbA and RcbB in enhancing biofilm formation. These findings suggested that the deletion of *rcbAB* impedes biofilm development following initial surface attachment and that RcbA and RcbB function co-operatively to enhance biofilm formation during the subsequent stages of biofilm maturation.

### RcbA and RcbB co-operatively inhibit *V. vulnificus* swimming motility

**Fig 3:**
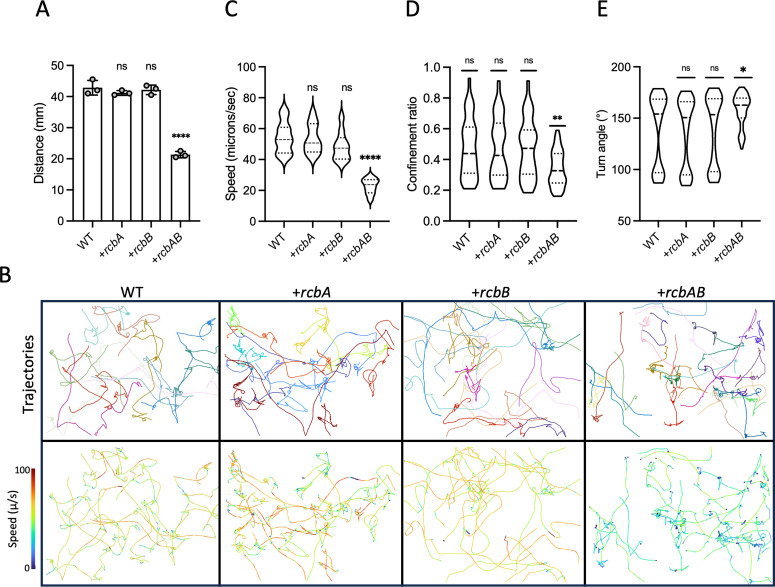
RcbA and RcbB co-operatively inhibit *V. vulnificus* swimming motility. A, plot of the swimming motility zones in soft agar by wildtype (WT) cells harboring the empty plasmid (pC2X6HT) or expressing (+) *rcbA, rcbB* or *rcbAB,* respectively. Bars indicate the respective mean values and error bars represent the standard deviation of triplicate assays. B, representative images of motility traces for the same samples in A. Different colored lines in the traces on top represent trajectories for individual cells and the corresponding traces colored by swimming speed are below. C-E, violin plots of the swimming speed, confinement ratio (net displacement/total distance traveled), and turn angles for the cells in B. Dashed horizontal lines denote the median, first and third quartiles for the data range from 100 tracks for each strain. Statistically significant differences (*p < 0.05, **p < 0.01, ****p < 0.0001; ns, no significant difference) relative to WT cells were determined by one-way ANOVA with Dunnett’s multiple comparisons post hoc test. Expression was induced with 10 µM IPTG.

Given the observed impact of *rcbAB* deletion and expression on *V. vulnificus* biofilm formation, the operon’s influence on swimming motility was investigated. Wildtype, Δ*rcbA*, Δ*rcbB*, and Δ*rcbAB* strains were inoculated into soft agar and motility zones were quantified after overnight incubation. Although an inverse correlation was observed between motility zone size and calcium concentration, there was no significant difference in motility between the strains at any condition tested (S2 Fig), suggesting that the effect of calcium on swimming motility was not *rcbAB* dependent. Swimming motility was also assessed for wildtype cells expressing *rcbA*, *rcbB*, or both genes simultaneously. Relative to wildtype cells carrying empty vector, cells expressing either *rcbA* or *rcbB* alone showed no significant change in motility zone size, while cells co-expressing *rcbAB* exhibited a nearly 2-fold decrease in motility zone diameter regardless of the calcium level (Figs 3A and S3), suggesting no direct calcium modulation of RcbAB activity.

To further characterize the motility phenotype, the swimming speed of individual cells was analyzed (Fig 3B). *Vibrio* species swim using a distinct, three-step pattern that includes forward runs, reversals of ~180°, and flicks of ~90° (run-reverse-flicks) that re-orient the cell [87,88]. Consistent with the population-level assays, the trajectories for cells expressing either *rcbA* or *rcbB* alone showed no significant difference in swimming speed, confinement ratio (a measure of the efficiency of movement of a cell from starting position to endpoint), or turn angles relative to control cells (Fig 3C–3E). In contrast, cells co-expressing *rcbA* and *rcbB* exhibited a 50% decrease in average swimming speed (from 53 µm sec^-1^ to 26 µm sec^-1^) and a 30% increase in average confinement (from an of 0.45 to 0.32). Moreover, the turn angles, which primarily fell into clusters of ~93° (flicks) and 167° (reversals) for wildtype cells, were skewed toward reversals, with fewer flicks. Collectively, these results suggested a negative synergistic effect of *rcbAB* expression on *V. vulnificus* motility behavior that restricted foraging.

### The TPR domain of RcbB represses its DGC activity and RcbA de-represses it

**Fig 4:**
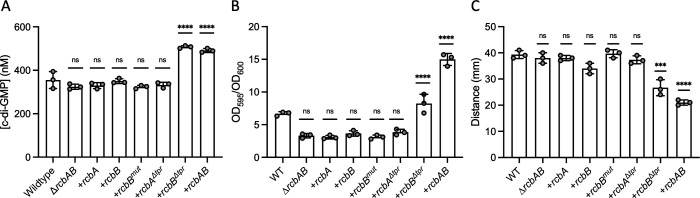
RcbA and RcbB co-operatively regulate c-di-GMP-dependent phenotypes. Plot of cellular c-di-GMP levels (A), biofilm formation (B) and motility in soft agar (C) of wildtype *V. vulnificus* (WT) and ∆*rcbAB* cells that carry the empty expression plasmid or express (+) *rcbA*, *rcbB*, *rcbB*^*mut*^ (GGAEF active site mutant), *rcbA*^Δ*tpr*^ (an RcbA TPR deletion mutant), *rcbB*^Δ*tpr*^ (an RcbB TPR deletion mutant) or the *rcbAB* operon. Bars show the respective mean values for each strain and error bars represent the standard deviation of triplicate assays. Statistically significant differences relative to wildtype were determined by one-way ANOVA with a Dunnett’s multiple comparisons post-hoc test (**p < 0.01, ***p < 0.001, ****p < 0.0001; ns, no significant difference). Expression was induced with 10 µM IPTG in A, 1 µM in B and 10 µM in C.

Since RcbB contained a predicted GGDEF domain, the cellular levels of c-di-GMP in *V. vulnificus* that expressed *rcbA*, *rcbB* or *rcbAB* was determined. Relative to wildtype, the level of cellular c-di-GMP was not altered by deletion of the operon, or in ∆*rcbAB* cells that expressed *rcbA*, *rcbB*, *rcbB*^*mut*^ (a catalytic mutant bearing the A-to-E mutation, GGAEF) or *rcbA*^∆*tpr*^ (which lacked the TPR domain) (Fig 4A). However, modest increases of 1.5-fold were observed for ∆*rcbAB* cells that expressed *rcbB*^∆*tpr*^ or co-expressed *rcbA* and *rcbB*, and this effect was not calcium dependent (S4 Fig). Biofilm formation by ∆*rcbAB* cells expressing *rcbA*, *rcbB*, *rcbB*^*mut*^ or *rcbA*^∆*tpr*^ was similar to ∆*rcbAB* cells carrying the empty vector (Fig 4B). Additionally, *rcbB*^*mut*^ was unable to promote biofilm formation or slow colony spread in the ∆*rcbB* background (S5 Fig), suggesting that the activity of RcbB was dependent on an intact catalytic motif. Notably, biofilm formation increased 2.5-fold for ∆*rcbAB* cells expressing *rcbB*^∆*tpr*^, although this was half that observed for cells expressing *rcbAB*. Likewise, the motility zones of cells expressing *rcbA*, *rcbB*, *rcbB*^*mut*^ or *rcbA*^∆*tpr*^ was similar to wildtype cells and, again, *rcbB*^∆*tpr*^ expression was less effective at decreasing motility (1.5-fold) than *rcbAB* expression (1.9-fold) (Fig 4C). These results suggested that *rcbB* coded for a functional DGC and the co-expression of *rcbA* enhanced RcbB activity, resulting in a synergistic increase in c-di-GMP production and concomitant effects on c-di-GMP related phenotypes.

**Fig 5:**
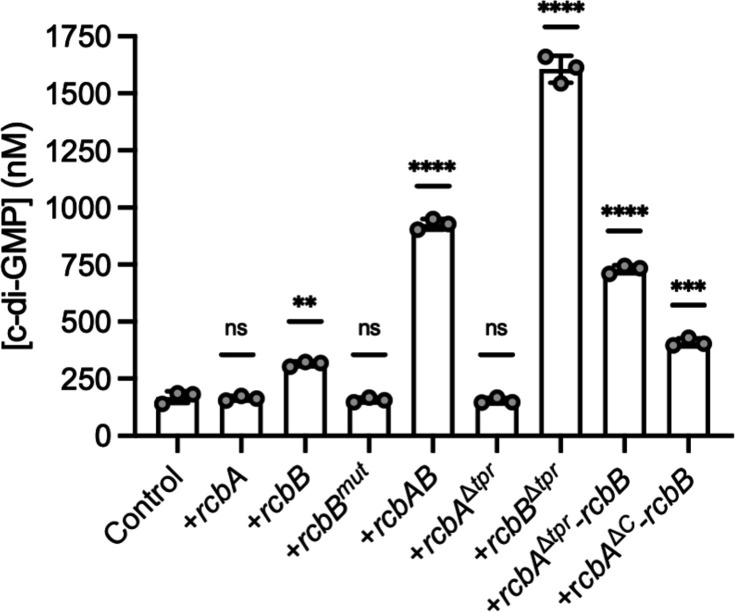
RcbA and RcbB co-operatively regulate c-di-GMP production and the TPR domain of RcbB suppresses its DGC activity. Plot of intracellular c-di-GMP levels in wildtype *E. coli* MG1655 cells that carry the empty expression plasmid (control) or express (+) *rcbA*, *rcbB*, *rcbB*^*mut*^ (GGAEF active site mutant), *rcbAB*, *rcbA*^Δ*tpr*^ (an RcbA TPR deletion mutant), *rcbB*^Δ*tpr*^ (an RcbB TPR deletion mutant), *rcbA*^Δ*C*^*-rcbB* (in which the C-terminus of RcbA is deleted), or *rcbA*^Δ*tpr*^*-rcbB.* (in which the TPR domain of RcbA is deleted). Bars show the respective mean values for each strain and error bars represent the standard deviation of triplicate assays. Statistically significant differences relative to the respective controls were determined by one-way ANOVA with a Dunnett’s multiple comparisons post-hoc test (**p < 0.01, ****p < 0.0001; ns, no significant difference). Expression was induced with 10 µM IPTG.

To mitigate possible complications from the plethora of *V. vulnificus* gene products predicted to participate in c-di-GMP turnover, cellular c-di-GMP levels were also measured in *E. coli* MG1655 cells, the motility of which, as observed in *V. vulnificus*, decreased in response to *rcbAB* expression (S6 Fig). Relative to control cells, the cellular level of c-di-GMP did not change in cells expressing *rcbA* alone (Fig 5). Although only a modest increase of 1.5-fold in the intracellular c-di-GMP level was observed for cells expressing *rcbB*, no increase was observed in cells expressing *rcbB*^*mut*^. Notably, the cellular c-di-GMP level of cells co-expressing *rcbA* and *rcbB* increased nearly 4-fold, analogous to the increase observed in *V. vulnificus*. Cells expressing *rcbA*^Δ*tpr*^ had c-di-GMP levels similar to control cells. In contrast, *rcbB*^Δ*tpr*^ expression resulted in an approximately 5-fold increase in c-di-GMP levels, suggesting that the TPR domain of RcbB strongly suppressed its DGC activity. Although *rcbA*^Δ*tpr*^*-rcbB* expression increased c-di-GMP levels 3-fold relative to control cells, this was less than observed for cells expressing *rcbAB*, and an even greater drop was observed for cells expressing an operon in which *rcbA* lacked its C-terminal region (*rcbA*^Δ*C*^*-rcbB*). Collectively, these results suggested that the TPR domain of RcbB inhibited its diguanylate cyclase activity and that RcbA relieved this inhibition using both its N-terminal TPR domain and C-terminal region, although we acknowledge that some of the differences in activity of the truncated versions of RcbA and RcbB could be due to changes in stability. Moreover, while removing TPR-mediated inhibition maximized c-di-GMP output by RcbB, this alone did not correlate with maximal phenotypic effect on biofilm formation and motility, and implied that the mechanism by which RcbA modulated RcbB activity extended beyond simple de-repression to, possibly, subcellular localization.

### RcbA and RcbB interact via their TPR domains

**Fig 6:**
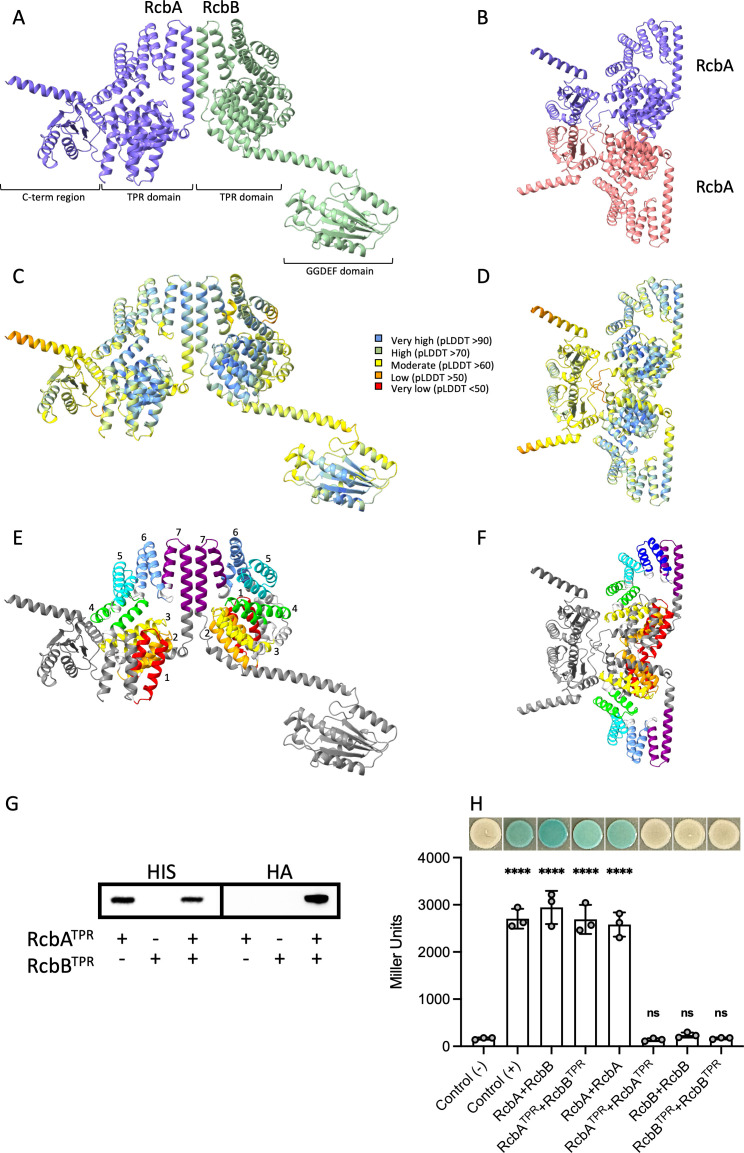
RcbA and RcbB interact via their TPR domains. AlphaFold 3 predicted structure of RcbA and RcbB complexes. Shown are a RcbA-RcbB heterodimer (A, C and E) and RcbA homodimer (B, D and F) colored by chain (A and B), per-atom confidence estimate (pLDDT; C and D) and rainbow for the TPR repeats (E and F). The N-terminal TPR domains of RcbA and RcbB are predicted to contain 7 TPR repeats each (numbered 1-7 and colored red, orange, yellow, green, cyan, light blue and purple, respectively). The C-terminus of RcbA and GGDEF domain of RcbB are colored grey. G, western blot of pull-downs using Ni-coated beads from extracts of *E. coli* producing either the HIS-tagged TPR domain of RcbA (RcbA^TPR^) or the HA-tagged TPR domain of RcbB (RcbB^TPR^) alone or together. Anti-HIS antibody (HIS) was used to detect RcbA^TPR^. The blot was stripped for subsequent detection of RcbB^TPR^ with anti-HA antibody (HA). Expression was induced with 50 µM IPTG. H, Bacterial Adenylate Cyclase Two-Hybrid (BACTH) assays of protein-protein interactions. RcbA, RcbB, RcbA^TPR^ and RcbB^TPR^ were fused to the C-terminus of *Bordetella pertussis* adenylate cyclase subunits T18 or T25. The plot shows β-galactosidase activity (Miller units) for the indicated protein combinations. Leucine zipper fusions to T18 and T25 served as a positive control (+), while empty plasmids were used as the negative control (-). Bars indicate the respective mean values and error bars represent the standard deviation of triplicate assays. Statistically significant differences (****p < 0.0001; ns, no significant difference) relative to the negative control were determined by one-way ANOVA with a Dunnett’s multiple comparisons post-hoc test. Representative images of the respective strains are shown above the plot.

The impact of RcbA on the activity of RcbB suggested a possible direct interaction between the proteins. AlphaFold 3 was used to predict the joint structure of a RcbA-RcbB complex (Fig 6A). The confidence of the overall predicted fold for the complex (Fig 6C) was high (pTM = 0.56), particularly for the 7 N-terminal TPR motifs of each protein (Fig 6E). A potential interaction surface between the final motif (TPR 7) of both proteins was predicted, as well as self-interaction by RcbA (Fig 6B, 6D and 6F).

Attempts to determine if RcbA and RcbB interacted *in vivo* using *V. vulnificus* cell extracts that contained HIS-tagged RcbA (RcbA), HA-tagged RcbB (RcbB) or both were unsuccessful, as the proteins produced (S7A Fig) were rapidly degraded following cell lysis; similar issues were encountered using extracts containing only the TPR domains of the proteins. Instead, *E. coli* cell extracts containing the HIS-tagged TPR domain of RcbA (RcbA^TPR^), the HA-tagged TPR domain of RcbB (RcbB^TPR^) or both were incubated with nickel-coated magnetic beads, and recovered proteins were analyzed by immunoblot. A band was observed with anti-HIS antibody for the two extracts that contained RcbA^TPR^, while no signal was detected for cells producing only RcbB^TPR^ (Fig 6G). The blot was then stripped and re-probed with anti-HA antibody. Notably, no signal was observed for extracts containing only RcbA^TPR^ or RcbB^TPR^, whereas a signal was observed for cells producing both proteins. Collectively, these results suggested that RcbA and RcbB interacted via their TPR domains.

Interaction between RcbA and RcbB was also assessed by Bacterial Adenylate Cyclase Two-Hybrid (BACTH) analysis. RcbA and RcbA^TPR^ were fused to the C-terminus of *Bordetella pertussis* adenylate cyclase subunit T18, and RcbB and RcbB^TPR^ were fused to adenylate cyclase subunit T25. Co-production of T18-RcbA and T25-RcbB resulted in blue colonies and β-galactosidase activity equal to the positive control, suggesting strong interaction between the two proteins (Fig 6H). Co-production of T18-RcbA^TPR^ and T25-RcbB^TPR^ also produced blue colonies and high β-galactosidase activity, suggesting that the interaction is mediated via their TPR domains. RcbA self-interaction was also observed, suggesting that it can form dimers or possibly higher order oligomers. However, interaction between T18-RcbA^TPR^ and T25-RcbA^TPR^ was not detected, arguing against self-interaction being mediated by the TPR domain of RcbA. White colonies and minimal β-galactosidase activity for cells producing T18-RcbB and T25-RcbB or T18-RcbB^TPR^ and T25-RcbB^TPR^ suggested that RcbB does not self-interact. Collectively, these results support the notion that RcbA and RcbB interact with each other via their TPR domains, while RcbA exhibits TPR-independent self-interaction.

### RcbA and RcbB localize to the flagellar pole

**Fig 7:**
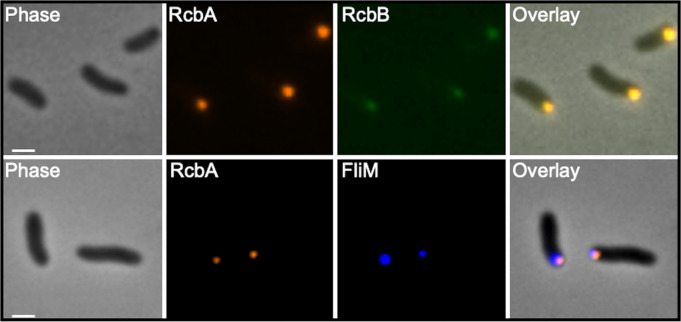
RcbA and RcbB localize to the flagellar pole. Representative phase, fluorescence and overlay images of ∆*rcbAB* cells expressing *rcbA-mRuby3* and *rcbB-mNeonGreen* (top panels) and ∆*fliM*∆*rcbA* cells expressing *rcbA-mRuby3* and *fliM-bfp* (bottom panels). Scale bars = 1 μm. Expression of *rcbA* and *rcbB* was induced with 10 µM IPTG and *fliM* was induced with 0.05% L-arabinose.

The previous data raised the possibility of subcellular localization of RcbA and RcbB in the cell. The positions of the proteins were monitored in ∆*rcbAB* cells using fluorescently tagged fusions (RcbA-mRuby3 and RcbB-mNeonGreen) that were functional and promoted biofilm formation at levels comparable to the wildtype proteins (S8 Fig). Both proteins localized to the same cell pole (Fig 7). Since co-expression of *rcbA* and *rcbB* co-operatively slowed swimming motility, we determined if RcbA and RcbB localized to the flagellar pole. FliM that was C-terminally tagged with blue fluorescent protein (BFP) was used as a marker of the flagellar pole. Co-expression of *rcbA-mRuby3* and *fliM-bfp* in a Δ*fliM*Δ*rcbA* background revealed polar fluorescent foci for the corresponding proteins at the same pole. Since RcbB localizes to the same pole as RcbA, collectively these data suggested that RcbA and RcbB localized to the flagellar pole.

### The TPR domain mediates localization of RcbA and RcbB to the cell pole

**Fig 8:**
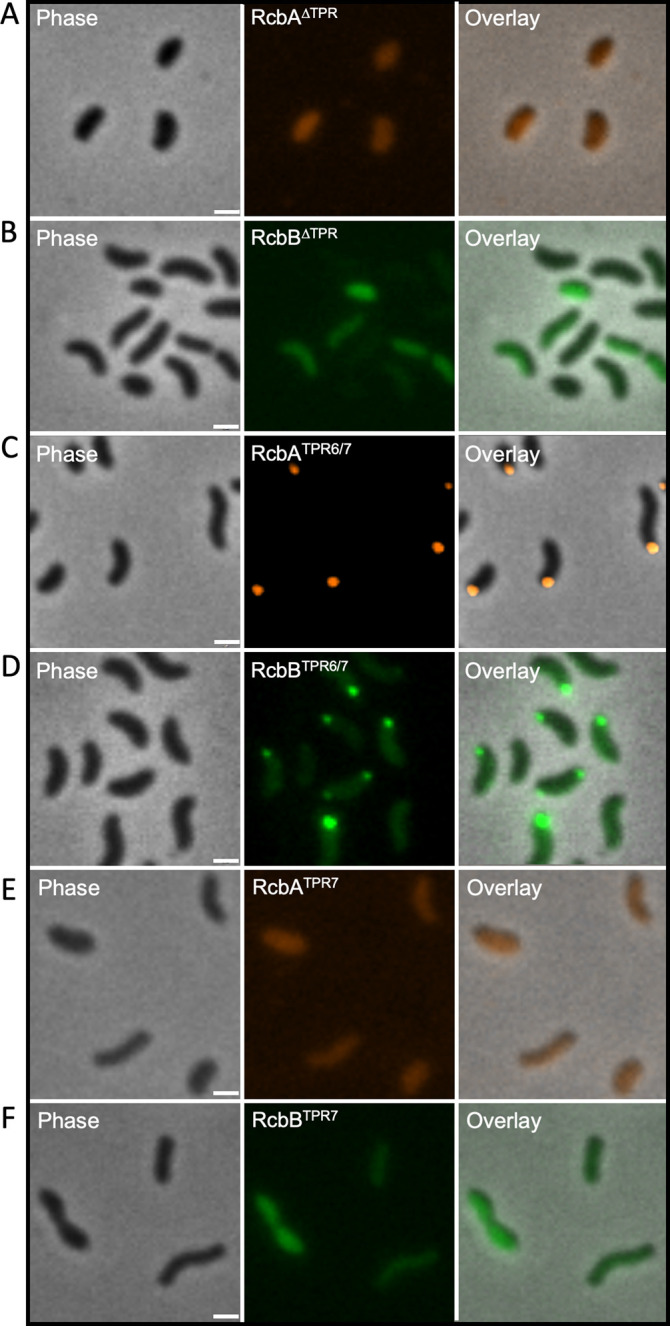
TPR6 and TPR7 of RcbA and RcbB are necessary for polar localization. From left to right, representative phase, fluorescence and overlay images of RcbA^∆TPR^-mRuby3 signal in ∆*rcbA* cells (A), RcbB^∆TPR^-mNeonGreen signal in ∆*rcbB* cells (B), RcbA^TPR6/7^-mRuby3 signal in the ∆*rcbA* cells (C), RcbB^TPR6/7^-mNeonGreen signal in ∆*rcbB* cells (D), RcbA^TPR7^-mRuby3 signal in the ∆*rcbA* cells (E) and RcbB^TPR7^-mNeonGreen signal in ∆*rcbB* cells (F). Scale bar = 1 μm. Expression was induced with 10 µM IPTG.

To elucidate the role of the TPR domain in the polar localization of RcbA and RcbB, fluorescently tagged variants lacking the TPR domain (RcbA^∆TPR^mRuby3 and RcbB^∆TPR^mNeonGreen), which were predicted to correctly fold and were produced in *V. vulnificus* (S7B and S7C Fig), were expressed in Δ*rcbA* and Δ*rcbB* cells, respectively, and their subcellular distribution was monitored. In contrast to the wildtype proteins, these truncated variants produced diffuse cytoplasmic signals (Fig 8A and 8B), suggesting that the TPR domain was needed for polar localization of both RcbA and RcbB. The AlphaFold 3-predicted structure suggested that TPR-7 mediated the interaction between RcbA and RcbB (Fig 6E). To test this hypothesis, the localization of RcbA and RcbB variants lacking TPR1-5 (RcbA^TPR6/7^-mRuby3 and RcbB^TPR6/7^-mNeonGreen) was determined. Fluorescence microscopy revealed that the truncated variants continued to localize to the cell pole (Fig 8C and 8D). However, further truncation to remove TPR6 (RcbA^TPR7^-mRuby3 and RcbB^TPR7^-mNeonGreen) resulted in diffuse cytoplasmic signals for both proteins (Fig 8E and 8F). These observations suggested that both TPR6 and TPR7 were needed for polar localization of RcbA and RcbB.

### Polar localization of RcbB and RcbA is not co-dependent but RcbA anchors RcbB in the absence of polar landmarks

**Fig 9:**
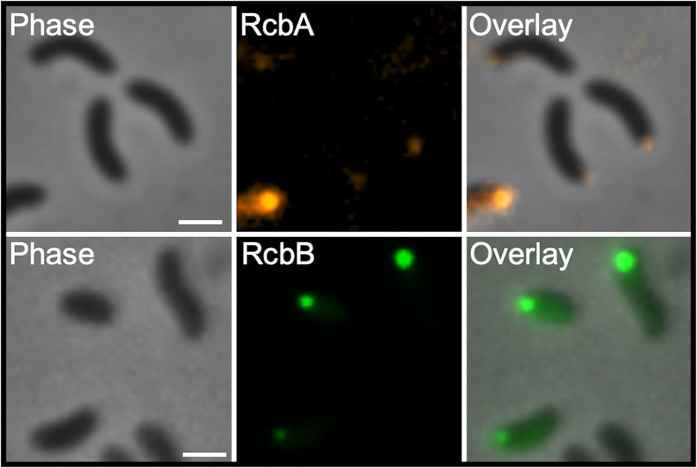
Polar localization of RcbA and RcbB is not co-dependent. Representative phase, fluorescence and overlay images of ∆*rcbAB* cells expressing *rcbA-mRuby3* (top panels) or *rcbB-mNeonGreen* (bottom panels). Scale bars = 1 μm. Expression was induced with 10 µM IPTG.

**Fig 10:**
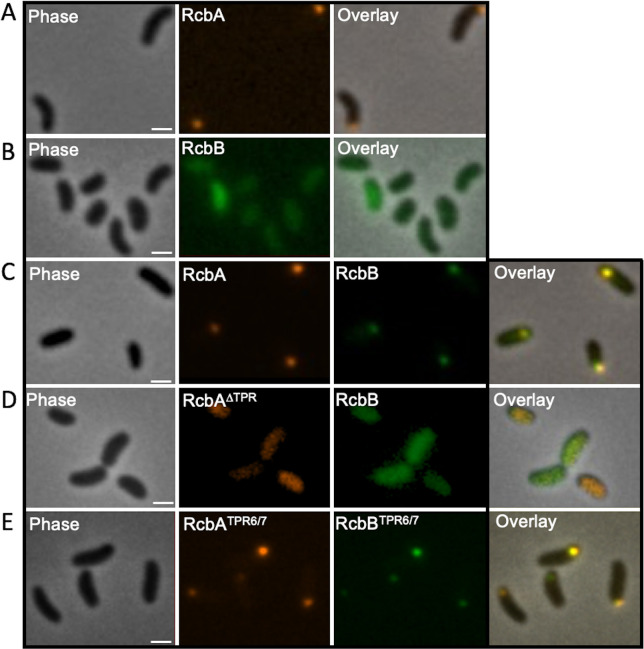
RcbA anchors RcbB to the pole in the absence of the polar landmark protein HubP. From left to right, representative phase, fluorescence and overlay images of RcbA-mRuby3 (A), RcbB-mNeonGreen (B), both (C), RcbA^∆TPR^-mRuby3 and RcbB-mNeonGreen (D) or RcbA^TPR6/7^-mRuby3 and RcbB^TPR6/7^-mNeonGreen signal (E) in ∆*hubP*∆*rcbAB* cells. Scale bar = 1 μm. Expression was induced with 10 µM IPTG.

To elucidate the interdependence of RcbA and RcbB for polar localization, the subcellular distribution of RcbA-mRuby3 and RcbB-mNeonGreen was separately monitored in Δ*rcbAB* cells. The signal for RcbA-mRuby3 remained polar in the absence of RcbB and the signal for RcbB-mNeonGreen was polar in the absence of RcbA (Fig 9). This suggested that RcbA and RcbB could independently localize to the cell pole without prior interaction. To investigate the role of polar landmarks in the localization of these proteins, the fluorescence patterns of RcbA-mRuby3 and RcbB-mNeonGreen were monitored in a Δ*hubP*Δ*rcbAB* background. HubP plays a crucial role in organizing and coordinating various cellular processes at the bacterial cell pole [89–92]. In this genetic context, RcbA-mRuby3 maintained its polar localization, whereas RcbB-mNeonGreen displayed a diffuse cytoplasmic distribution (Fig 10A and 10B). These results indicated that the polar localization of RcbB, but not RcbA, was dependent on HubP. Cells lacking *hubP* retained the ability to produce polar flagella (S9 Fig). To determine if RcbA and RcbB localization was dependent on polar flagellar synthesis, their subcellular position was monitored in a ∆*flrA*∆*rcbAB* background. FlrA is a master regulator of flagellar gene expression in *Vibrio* species [93–95] and its deletion results in cells that lack flagella (S9 Fig). The same localization pattern for RcbA-mRuby3 and RcbB-mNeonGreen that was observed in Δ*hubP*Δ*rcbAB* cells was observed in ∆*flrA*∆*rcbAB* cells (S10A and S10B Fig). Notably, co-expression of RcbA mitigated the effects of HubP or FlrA deletion on polar localization of RcbB in the ∆*hubP*∆*rcbAB* and ∆*flrA*∆*rcbAB* backgrounds (Figs 10C and S10C). However, an RcbA variant lacking its TPR domain failed to anchor RcbB at the cell pole (Fig 10D). Since RcbA containing only TPR6/7 successfully tethered RcbB also bearing TPR6/7 to the pole in ∆*hubP*∆*rcbAB* cells (Fig 10E), this suggested that these repeats were needed for RcbA-mediated polar anchoring of RcbB. Thus, polar localization of RcbA and RcbB was not co-dependent, but RcbA anchored RcbB to the pole in the absence of the polar landmark protein HubP and flagella.

### Phylogenetic distribution of *rcbAB*

Among the γ-proteobacteria, homologs of the *rcbAB* operon were identified in 73 out of 91 distinct *Vibrio* species represented in publicly accessible complete genome assemblies, encompassing a total of 583 out of 617 genomes (S2 Table). Since only one or two genome assemblies were available for some species, sequencing of additional isolates may yet reveal extant strains with *rcbAB* homologs for these species (e.g., the genomes of two *V. alginolyticus* isolates lack *rcbAB*, whereas 43 code for it). In most instances (96%), the operon was situated on the smaller of the two chromosomes within each species, and in all cases, *rcbA* was found upstream of *rcbB*. The operon was also detected in other genera within the Vibrionaceae, specifically *Allivibrio* and *Thaumasiovibrio*.

**Fig 11:**
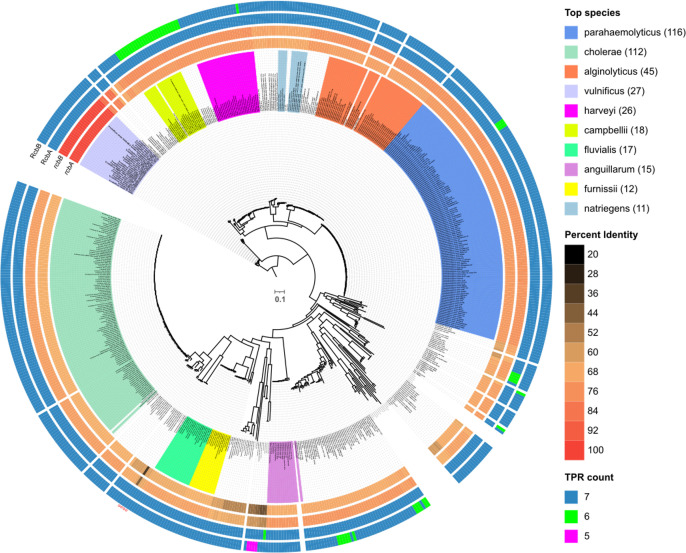
Phylogenetic distribution of *rcbAB* among *Vibrio* species. From inside-out, Maximum Likelihood tree (rooted with *V. vulnificus*) based on 133 common core genes from 617 complete *Vibrio* genome assemblies, with branch lengths representing substitutions per site. Clades are color-coded for the ten most frequently represented *Vibrio* species, with the number of genomes harboring *rcbAB* indicated in parentheses. Taxa names are labeled on the tree. The inner two color-gradient tracks show percent identity to *rcbA* and *rcbB* from *V. vunificus* ATCC27562 of orthologs from the respective strain, while the outer two tracks indicate the number of tetratricopeptide repeats (TPR count) identified in the corresponding proteins.

The homology of RcbA orthologs varied from 100% in *V. vulnificus* to a mere 28.5% in *V. cincinnatiensis*, while the homology of RcbB orthologs ranged from 100% to 43.6% (Fig 11). Each species possessed a single copy of the operon, apart from *V. cincinnatiensis*, which harbored two copies located more than 230 kb apart on the same chromosome. While the products of one of the operons shared approximately 60% overall identity to those of *V. vulnificus*, the other, despite exhibiting only about 36% overall identity, retained TPR and DGC domain features, suggesting a common progenitor that evolved by gene duplication and potential sub-functionalization of the paralogs in this species.

Notably, despite significant variability in sequence, RcbA homologs consistently displayed a configuration comprising 7 tetratricopeptide motifs (TPRs). Only three species (*V. europaeus*, *V. palustris*, and *V. tubiashii*) harbored homologs with six TPRs. The majority of RcbB homologs also exhibited a configuration of seven TPRs; however, greater variability was observed, with at least ten species possessing homologs with six TPRs (*V. atlanticus*, *V. bathopelagicus*, *V. campbellii*, *V. cortegadensis*, *V. gallaecicus*, *V. owensii*, *V. pectenicida*, *V. pelagius*, and *V. syngnathi*), alongside two species that contained homologs with only five TPRs (*V. tritonius* and *V. poteresiae*).

A search among non-Vibrionaceae genomes yielded 257 chromosomes encoding 341 *rcbAB* homologs in the families Alteromonadaceae (genera *Alteromonas* and *Motilimonas*) and Psychromonadaceae (genus *Psychromonas*), 53 of which had more than one copy and 55 of which had *rcbA* positioned downstream of *rcbB.* Among β-proteobacteria, homologs of the *rcbAB* operon were found within the Chitinibacteraceae, represented by genera such as *Andreprevotia*, *Chitinibacter*, *Chitinilyticum*, *Chitiniphilus*, *Deefgea*, *Formivibrio*, and *Jeongeupia* (S3 Table). Most homologs shared 10–40% identity with *rcbA* and *rcbB* of *V. vulnificus* and gene order was preserved across most genera; however, *rcbB* was positioned upstream of *rcbA* in *Jeongeupia* and some *Chitinibacter* species (e.g., *C. bivalviorum* strain 2T18 had two *rcbAB* and four *rcbBA* homologs). The conservation of the operon across diverse bacterial taxa implies that the functions of RcbA and RcbB are fundamentally important for the organisms in which they are found.

## Discussion

**Fig 12:**
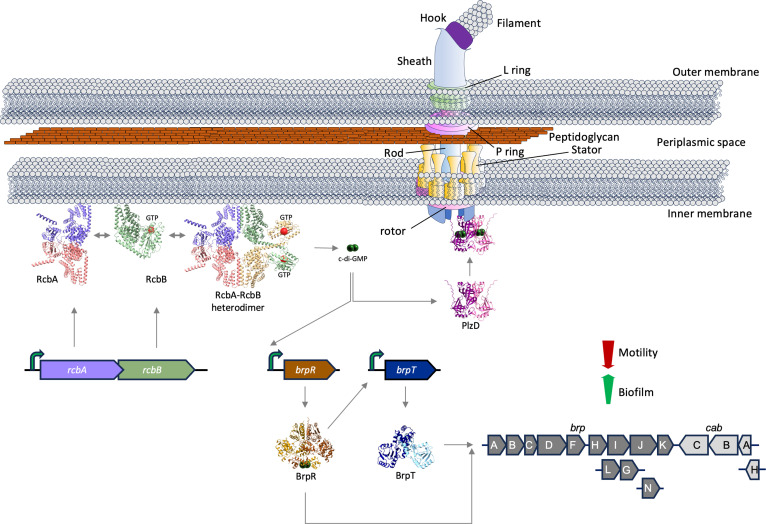
Model for subcellular localization and interaction of RcbA and RcbB in regulating bacterial biofilm formation and motility. Expression of the bicistronic operon produces RcbA (purple) and RcbB (green), which can independently localize to the flagellar pole. RcbA can self-assemble into homomultimers (purple/red), while RcbB cannot; however, RcbA may facilitate the formation of higher-order oligomers involving RcbB (green/gold) that are functionally important, since DGCs typically function as protein dimers. The N-terminal TPR domain of RcbB strongly inhibits its C-terminal DGC activity, keeping it in an autoinhibited state. RcbA anchors RcbB to the flagellar pole for optimal phenotypic effect and relieves RcbB’s autoinhibition. Elevated c-di-GMP production increases expression of *brpR* (brown), which encodes a transcriptional activator and potential c-di-GMP effector. BrpR in turn upregulates expression of the regulator encoded by *brpT* (blue). This cascade induces the synthesis of biofilm matrix components such as BRP exopolysaccharides (EPS) and calcium-binding proteins (dark and light grey, respectively), promoting biofilm development. Additionally, c-di-GMP may be sensed by effector proteins such as PlzD, which localizes to the flagellar pole and reduces the swimming speed and turn frequency of motile cells.

Biofilm formation is an ancient and universal survival mechanism [23,96]. c-di-GMP is a key regulator of biofilm formation [33,97], increases in which generally enhance EPS production and biofilm formation while repressing flagellar-mediated swimming motility and virulence gene expression. Here, we identify *rcbAB*, a two-gene operon coding for proteins that partner to regulate c-di-GMP-dependent phenotypes in *V. vulnificus* and provide insight into the functional dynamics of RcbA and RcbB in the cell. The reduction in post-attachment biofilm biomass of a Δ*rcbAB* strain supports a significant role for *rcbAB* in biofilm maturation. Localization of both proteins to the flagellar pole suggested a role for RcbA and RcbB in motility regulation, and co-expression of *rcbAB* suppressed motility; however, its deletion had no impact on the phenotype. This suggests that the operon primarily influences biofilm formation, while perhaps playing an ancillary role in regulating swimming motility. RcbA contains an N-terminal TPR domain and a structured C-terminal region of unknown function, while RcbB possesses an N-terminal TPR domain and a C-terminal diguanylate cyclase domain. A recent study in *V. parahaemolyticus* described the function of two separately coded DGCs with predicted periplasmic TPR domains that additively repress the expression of genes required for swarming motility [98]. Both proteins required an intact catalytic diguanylate cyclase motif to function, however, it was not determined if the TPR domains of the DGCs affected their subcellular location or activity, nor were their interacting targets, if any, identified. Removing the TPR domain of RcbB maximized its c-di-GMP output, but this alone did not correlate with maximal phenotypic effect. RcbA and RcbB interact via their N-terminal TPR domains, with RcbA both de-repressing the diguanylate cyclase activity of RcbB and, notably, localizing it to the flagellar pole. The observation that RcbA anchors RcbB at the pole in the absence of polar landmarks such as HubP and flagella suggests a hierarchical regulatory mechanism where RcbA may serve as a scaffold for RcbB. It also suggests that the spatial organization of the RcbA-RcbB complex in the cell is important for function. It is conceivable that polar localization of RcbB’s diguanylate activity generates a high-c-di-GMP microenvironment in the vicinity of the flagellum that can be sensed by nearby proteins so that bacteria can fine tune physiological responses based on environmental cues. The proposed model (Fig 12) illustrates how the interaction and subcellular localization of RcbA and RcbB might regulate biofilm formation and swimming motility by modulating local c-di-GMP levels. The N-terminal TPR domain of RcbB strongly inhibits its C-terminal DGC activity, keeping it in an autoinhibited state. Since DGCs typically function as protein dimers or higher order oligomers [99–101], RcbA, which can self-assemble into homomultimers, facilitates the formation of heterotetramers or higher-order oligomers involving RcbB that are important for de-repression of its diguanylate cyclase activity (S11 Fig). The N-terminal TPR domain of RcbA anchors RcbB to the flagellar pole and, co-operatively with its C-terminal region, relieves RcbB’s autoinhibition to maximize the phenotypic impact of elevated, localized c-di-GMP concentrations. Elevated c-di-GMP levels increase expression of *brpR*, which encodes a transcriptional activator and potential c-di-GMP effector [64,65,102]. BrpR in turn upregulates *brpT* expression and together these regulators induce the synthesis of biofilm matrix components such as BRP EPS and calcium-binding proteins to promote biofilm development. Additionally, c-di-GMP produced by RcbB may be sensed by effectors such as PlzD, the positioning of which at the flagellar pole in slow moving, initially adherent and mature biofilm cells, is regulated by c-di-GMP [69]. This outcome alters the foraging behavior of cells by slowing swimming speed and decreasing the number of directional changes, ultimately limiting exploration of the surrounding 3D space. Collectively, these events facilitate the transition from a planktonic to a sessile bacterial lifestyle.

Spatial regulation by targeting diguanylate cyclase activity to the cell pole of bacteria has profound implications for bacterial physiology, as heterogeneity in cellular c-di-GMP levels can lead to diverse phenotypic outcomes within a clonal population, enabling bacteria to better adapt to varying environmental conditions. DGCs, such as YfiN in *Escherichia coli*, have been shown to relocate to the division site under stress conditions, where they inhibit cell division by preventing septal peptidoglycan synthesis [103]. The DGC PleD exhibits a cell cycle-dependent localization pattern in *Caulobacter crescentus*. In swarmer cells, PleD remains in an inactive state but undergoes activation during the swarmer-to-stalked cell transition [101]. Its activity is intricately linked to its spatial redistribution to the stalked pole of the cell, suggesting that PleD activates proximal downstream effectors involved in pole remodeling [104]. In *Pseudomonas aeruginosa*, the DGC DgcP interacts with FimV, a polar peptidoglycan-binding protein, influencing local c-di-GMP production at the cell pole and type IV pilus assembly [105].

Polar localization of RcbA appears to be an intrinsic characteristic, while the localization of RcbB in the absence of RcbA is contingent upon the presence of HubP or polar flagella. These localization dynamics bear resemblance to the interactions of FlhF and FlhG, two proteins that regulate flagellar assembly and positioning in *Vibrio* species. Specifically, the polar localization of FlhF facilitates flagellar formation and establishes its spatial orientation, whereas FlhG modulates the quantity of flagella produced by interacting with FlhF to inhibit its polar localization, thereby regulating flagellar number [106]. The coordinated activities of these proteins are crucial for the precise determination of flagellar quantity and placement. Historically, it was posited that polar localization of FlhF was intrinsic [107,108], while that of FlhG was dependent on HubP [91,109]. However, recent findings have demonstrated that FlhF can be recruited to the cell pole by the small integral membrane protein, FipA [89]. At this juncture, we cannot exclude the possibility that a similar mechanism may govern polar localization of RcbA and future investigations will aim to ascertain whether RcbA possesses the capability of independent polar localization.

The TPR motif has been found in organisms ranging from bacteria to humans [74]. TPR motif-containing proteins act as scaffolds for the assembly of different multiprotein complexes and play important roles in various cellular processes and pathogenesis [72,110]. Most studies describe TPR domains binding to peptide motifs or other protein regions [73,111], and although multiple TPR-containing proteins may indirectly interact within the same complex, no direct evidence exists of two proteins interacting specifically via their TPR domains (i.e., a TPR-TPR interaction). RcbA and RcbB, and most of their orthologs, were found to harbor 7 repeats in their TPR domains and interacted via these domains to form a heterocomplex. To our knowledge, this is the first evidence supporting an interaction between the TPR domains of two proteins, challenging the conventional paradigm in which only one protein partner bears the domain. Moreover, TPR 6 and 7 were necessary for interaction between RcbA and RcbB, and for their localization to the cell pole. It is tempting to speculate that TPR1-5 of RcbA and RcbB interact with other proteins as part of a larger assemblage at the cell pole. Within the periplasm of *E. coli*, the TPR domain of the outer membrane porin PgaA interacts with the deacetylation domain of PgaB to form a complex at the outer membrane that increases the deacetylation and glycoside hydrolase activities of PgaB as poly-β(1,6)-N-acetylglucosamine is transported to the cell surface [112]. Additional studies demonstrated that only the TPRs of PgaA that were proximal to its porin moiety interacted with PgaB, leading the authors to propose that its distal TPR motifs spanned the periplasm and interacted with inner membrane components of the secretion apparatus [113]. Additionally, the C-terminal region of RcbA, which bears no identifiable domains, is predicted to adopt extensive secondary structure. Participation of both domains of RcbA in modulating the diguanylate cyclase activity of RcbB hints at a sophisticated regulatory mechanism and nuanced level of control that may be pivotal for RcbA-RcbB function. Further investigation is needed to elucidate the precise nature of the molecular interactions between RcbA and RcbB, as well as their potential association with other proteins at the cell pole.

The *rcbAB* operon is widely distributed among pathogenic *Vibrio* species [4,8], symbionts [114–118], other genera within the Vibrionaceae [5], and beyond. This broad distribution suggests an evolutionary advantage conferred by this operon, likely due to its role in biofilm formation and motility - critical factors for survival in diverse aquatic environments. In support of this notion, GefB, a DGC that was recently reported to regulate biofilm formation, swarming and swimming motility in *V. parahaemolyticus* [119], is a homolog of RcbB and is transcribed from a bicistronic operon that also codes for a homolog of RcbA. It is likely that the functions of RcbA and RcbB have been maintained across many of these species, however, the variability observed among more distant homologs raises the possibility of functional divergence and adaptation among some, warranting further investigation into how these variations impact the ecological fitness of the species in which they are found.

The *V. vulniﬁcus* genome encodes nearly 100 proteins predicted to make, break, and bind c-di-GMP [120,121], but relatively little is known regarding the environmental signals that regulate c-di-GMP levels and bioﬁlm formation in response to changing environmental conditions. Calcium is a key environmental signal inducing c-di-GMP accumulation and upregulating exopolysaccharide, pilus, and matrix protein expression in *V. vulnificus* [56–60]. However, important aspects of calcium-dependent mechanisms that govern biofilm formation and motility in *V. vulnificus* remain unresolved and warrant investigation to address *Vibrio* biofilm challenges in clinical and environmental contexts. Future studies should focus on identifying environmental factors (e.g., cell density, temperature, nutrient availability) and signaling pathways that modulate RcbAB function and c-di-GMP dynamics. Apropos, the homologous *V. parahaemolyticus vpa1477-gefB* operon is reportedly regulated by the quorum-sensing regulators AphA and OpaR [119].

## Materials and methods

### Media and strains

LB, LB agar, Peptone and Yeast Extract were purchased from BD Difco and Instant Ocean was purchased from Instant Ocean. Antibiotics and additives were purchased from Sigma and used at the following concentrations: rifampin (Rf), 100 µg ml^-1^; kanamycin (Km), 160 µg ml^-1^; chloramphenicol (Cm), 25 µg ml^-1^ for *E. coli* and 2 µg ml^-1^ for *V. vulnificus*; ampicillin (Ap), 100 µg ml^-1^; gentamicin (Gm), 10 µg/ml for *E. coli* and 35 µg ml^-1^ for *V. vulnificus*; trimethoprim (Tp), 10 µg ml^-1^; 5-bromo-4-chloro-3-indolyl-β-D-galacto-pyranoside (X-Gal), 100 µg ml^-1^; L-arabinose (ara), 0.05%; and CaCl_2_ (15 mM); isopropyl-β-D-thiogalactopyranoside (IPTG), see figure legends for concentrations used in each experiment. Instant Ocean was used at 20 parts per thousand (IO20) and was supplemented with 0.5% yeast extract and 1% peptone (IO20YP) for growth of *V. vulnificus*. A spontaneous Rf resistant isolate of *V. vulnificus* strain ATCC27562 was used as the parental strain and *E. coli* S17.1λπ was used for plasmid transfer to *V. vulnificus* by conjugation.

### Construction of the *P*_*brpA*_*lacZ* and *P*_*cabA*_*lacZ* reporter plasmids and screening of a transposon library

We previously constructed a 325 bp *P*_*brpA*_ PCR product that was fused to the *E. coli lacZ* (*lacZ*^*Ec*^) and inserted into pTX1CebgA by Gibson assembly [56]. A similar construct was generated using a 271 bp *P*_*cabA*_ PCR product. Plasmids were maintained in *E. coli* S17.1λπ and conjugated to *V. vulnificus* for integration into *ebgA* (a cryptic *lacZ*). Transconjugants were selected on LB Rf Cm plates. To monitor *β*-galactosidase activity in *V. vulnificus* without interference due to expression of its endogenous *lacZ* (*lacZ*^*Vv*^), the reporter plasmids were conjugated to a *V. vulnificus* strain bearing a markerless deletion of the promoter for *lacZ*^*Vv*^ that was created using the pRE112 sucrose counterselection plasmid [122].

### Tn mutagenesis

The mini-Tn10 delivery vector pNKTXI-SceI [123] was used for transposon mutagenesis as previously described [56]. Briefly, *E. coli* strains carrying the plasmid were used as donors in mating experiments to transfer the plasmid to *V. vulnificus* strains bearing the *P*_*brpA*_*lacZ* reporter via conjugation. After overnight growth, transconjugants were selected by streaking onto LB plates with Rf, X-Gal, Cm, Km and CaCl_2_. After 48 hours of growth, white colonies representing loss of *brp* expression in the presence of CaCl_2_ (a total of 30,000 mutants were screened) were re-screened on the same type of selective plates. Verified mutants were pooled, collective genomic DNA was prepared and sent for whole-genome sequencing (Eurofins). Geneious (Biomatters) was used to map the reads to the mini-Tn10 reference sequence and recover flanking *V. vulnificus* genomic sequences that were mapped back against the whole genome of strain ATCC 27562 [121].

### Mutant construction and complementation

Allelic replacement of target genes was achieved as follows: PCR fragments corresponding to 1 kb upstream and downstream of the target gene and bearing overlaps with a central Tp, Km or Cm antibiotic resistance cassette were amplified and ligated to the cassette by Gibson reaction. Recipient strains carrying the pMMB-TfoX expression plasmid [124] were induced overnight in LB containing Ap and IPTG at 30°C. A 10 µl aliquot of cells was added to 500 µl of IO20 containing IPTG and 25 µl of the Gibson reaction. The transformation mixture was incubated statically overnight at 30°C. The next day, 1 ml of LB was added to the tube and cells were allowed to outgrow for 3 hours before plating on selective LB containing the appropriate antibiotic. Allelic replacement of the target gene was confirmed by PCR. For complementation, *rcbA*, *rcbB* and variants were amplified alone or as an operon and cloned into pC2X6HT [62] by Gibson assembly and expression was induced with IPTG; *fliM* was cloned into pSU38GT [61] and expression was induced with L-arabinose. Plasmids were conjugated from *E. coli* S17.1λπ to *V. vulnificus* and transconjugants were selected on LB Rf Ap plates.

### Colony morphology assays and crystal violet staining of biofilms in 96-well plates

For colony morphology assays, strains were grown overnight with agitation in LB containing the appropriate antibiotics at 30°C. Then 3 μl of each bacterial culture was spotted on LB agar plates containing antibiotics. Plates included X-Gal for visualization of *P*_*brpA*_*lacZ* expression. Images were captured after 48 hours using a Leica MS5 dissecting scope equipped with a Leica DC300F camera.

For crystal violet staining, strains were grown in IO20YP that contained the appropriate antibiotics at 30°C with shaking until they reached an OD_600_ of 1. Cultures were adjusted to 1x10^6^ CFU ml^-1^ in fresh media, and 150 µl was inoculated in triplicate into 96-well microtiter plates. Microtiter plates were incubated statically for 20 hours at 30°C. Following measurement of the planktonic cell density at OD_600_ on a BioTek Synergy HT microplate spectrophotometer, 50 µl of 0.4% crystal violet (CV) solution was added to each well. After 30 min, the stained wells were washed three times with distilled water and the remaining dye was resolubilized in 200 µl of 30% acetic acid. The OD_595_ of each well was then recorded and the ratio of OD_595_/OD_600_ was calculated to determine the relative level of biofilm formation. All assays were done in triplicate.

### Biofilm development in microfluidic chambers

Polydimethylsiloxane (PDMS)-glass flow cell devices containing eight 40 x 5 x 1-mm chambers were fabricated and sterilized [125]. Mid-log-phase wildtype cells (OD_600_ of 0.1) grown in IO20YP were seeded into separate flow cell chambers. Control chambers for background fluorescence contained medium only. Initial attachment (no flow) proceeded for 20 min, followed by a flow rate of 0.25 ml min^-1^ in IO20 for 16 hours. The chambers were then flooded with the same medium containing 2.5 µM Syto21 (Molecular Probes) for 10 min. Biofilm images and z-stacks (20 x 1 µm slices) were captured with an Olympus IX83 microscope using a UPLSAPO 40x silicon oil immersion objective (NA, 1.25; WD, 0.3 mm). Background fluorescence was subtracted from each sample, and quantitative analysis to determine biomass was performed using cellSense (Olympus) and Comstat [126,127]. Data from three biological replicates were analyzed for each strain. The images presented are from a single representative experiment.

### Soft-agar swimming assays

*V. vulnificus* cells were grown overnight in IO20 that contained the appropriate antibiotics and inducers at 30°C with agitation. Bacterial culture (2 μl) was inoculated into semisolid IO20 agar (0.3%) plates supplemented with 1% Tryptone and included the appropriate antibiotics and inducers. Plates were incubated overnight at room temperature and the swimming diameter of each strain was measured. All assays were done in triplicate.

### Motility tracking

The motility of early-exponential (OD_600_ of 0.1) *V. vulniﬁcus* cells that were grown in IO20YP, washed, and resuspended in IO20 was recorded by dark-ﬁeld microscopy on an Olympus IX83 microscope using a 20x ELWD objective. A stack of at least 150 frames (100 ms exposure time) was recorded for each sample and the movement of 15 randomly sampled motile cells per stack was traced using the MTrackJ47 plugin in Comstat [126,127]. Data from three stacks of three biological replicates were analyzed for each strain. Traces shown are from a single experiment. Violin plots were created with Prism (GraphPad).

### Quantification of cellular c-di-GMP concentrations

Intracellular c-di-GMP concentrations were determined with the LUCERNA cyclic-di-GMP Assay Kit (Lucerna, Brooklyn, NY, United States) according to the manufacturer’s instructions. The same procedure was used for both *E. coli* MG1655 and *V. vulnificus*. Briefly, cells expressing *rcbA*, *rcbB*, *rcbAB* or their derivatives from pC2X6HT were grown overnight at 37°C with shaking in LB containing the appropriate antibiotics. Samples were diluted 100x into fresh media containing 10 µM IPTG, grown to an OD_600_ of 1.0 and kept on ice for 30 minutes. The samples were then diluted 1:10 in RNase-free water. Bacterial culture (50 μl) was transferred in triplicate into a 96-well black flat-bottom microtiter plate and 150 μl of reaction mix (50 μl 4X c-di-GMP assay (CA) buffer, 50 μl 4X bacterial compatibility (BC) reagent, 20 μl 10X fluorophore, 10 μl 20X c-di-GMP sensor, and 20 μl RNase-free water) was added to each well. The plate was incubated for 22 hours at room temperature in the dark. After incubation, readings were taken using a BioTek Synergy HT1 microplate reader with excitation set at 469 nm and emission at 501 nm.

### Co-immunoprecipitation assays

Motile *E. coli* DH5α cells that expressed *rcbA* or *rcbB* alone or together from pC2X6HT as 6-HIS and HA-tagged fusions, respectively, were used for co-IP assays. Cells were grown overnight in 5 ml LB Ap at 37°C. The next day, cells were diluted 1:100 in 50 ml of fresh media containing IPTG and grown to an OD_600_ of 0.3. Cells were pelleted and washed twice with 10 ml of ice-cold water. Pellets were resuspended in B-PER Reagent (Thermo Scientific) according to the manufacturer’s instructions and the extracts were centrifuged at 13,000 rpm for 5 minutes to pellet cellular debris. 50 µL of washed HisPur Ni-NTA magnetic beads (Thermo Scientific) was added to the supernatant, which contained the extracted proteins, and the samples were incubated with gentle rolling at room temperature for 30 min. The beads were then washed five times with B-PER to remove non-specifically bound proteins and contaminants. Bound proteins were eluted with buffer containing 250 mM imidazole and separated on NuPAGE 4–12% Bis-Tris pre-cast protein gels (Invitrogen). Western Blot analysis used anti-HIS (BioRad) and anti-HA (Thermo Scientific) HRP antibodies for detection of RcbA and RcbB, respectively.

### Fluorescence microscopy

Plasmid expressing mRuby3, mNeonGreen and BFP fusion proteins were conjugated to the indicated *V. vulnificus* strains and expression was induced with IPTG and/or L-ara. Images of individual cells were captured after cells were mounted on low melting point agarose pads. Images were captured on an Olympus IX83 inverted microscope using a 100 × 1.3 numerical aperture phase contrast objective. Phase contrast and fluorescence images were obtained with a Hamamatsu ORCA-R2 digital charge-coupled device camera, and the light source was the X-cite 120 light-emitting diode (Lumen Dynamics, Mississauga, ON, Canada). Emission filters were purchased from Chroma Technology (Bellows Falls, VT). The specific emission filters were DAPI-5060C-OMF [excitation (EX) filter, 377/50 nm; emission (EM) filter, 447/60 nm; dichroic mirror (DM), 409 nm], GFP-3035D-OMF (EX filter, 473/31 nm; EM filter, 520/35 nm; DM, 495 nm), and mCherry-B-OFF (EX filter, 562/40 nm; EM filter, 641/75 nm; DM, 593 nm). Data from three biological replicates were analyzed for each strain. Images were processed using cellSense software (Olympus) and those presented are from a single representative experiment.

### Bacterial-two-hybrid (BACTH) experiments

Two test plasmids (pUT18 and pKNT25) encoding complementary adenylate cyclase fragments fused to full-length RcbA, RcbB or their domains of interest were transformed into *E. coli* BTH101 cells. The resulting strains were grown overnight in LB that contained the appropriate antibiotics and inducers at 30°C with agitation. Then 3 μl of each bacterial culture was spotted on LB agar plates containing 40 μg ml^-1^ of X-gal and appropriate antibiotics. The plates were then incubated for 24–48 hours at 30°C and protein interactions were evaluated by both colony growth and development of blue coloration resulting from functional complementation of the split adenylate cyclase protein.

### β-Galactosidase assays

Quantification of β-galactosidase activity was carried out following the PopCulture β-gal assay protocol with the following amendments. Briefly, bacteria were grown overnight at 30°C in LB medium with 50 µM IPTG. Bacterial culture (20 μl) was transferred in triplicate into a black 96-well flat-bottom microtiter plate and 180 μl of reaction mix was added to each well (final concentrations of 60 mM Na_2_HPO_4_, 40 mM NaH_2_PO_4_, 10 mM KCl, 1 mM MgSO_4_, 0.1 mg ml^-1^ lysozyme, 6 mg ml^-1^ *o*-nitrophenyl-β-D-galactopyranoside (ONPG), and 4% PopCulture reagent (Millipore). Blank wells contained 20 μl LB medium. The plate was quickly transferred to a BioTek Synergy HT1 microplate reader set to 30°C. OD_600_ and OD_420_ readings were taken every 60 s for 30 min. The microtiter plate was shaken at 500 rpm (double orbital) between readings to ensure proper mixing and sample lysis. The slope of OD_420_ readings over time (OD_420_ per minute) and the initial OD_600_ reading were used to calculate Miller units as previously described [128] using the following formula: Miller units = (5,000 × OD_420_ min^−1^)/OD_600_. Data from three technical replicates of three biological replicates were collected for all experiments.

### Structural modeling

The structure of RcbA and RcbB complexes were predicted using AlphaFold 3 [129]. Five homology models were generated for each protein, and the model with the best overall agreement score was analyzed further. ChimeraX [130] was used for molecular visualization.

### Statistical analyses

Statistical signiﬁcance was determined by Student's t-test (two-tailed distribution with two-sample, equal variance) when directly comparing two conditions or one-way analysis of variance (ANOVA) followed by pairwise comparisons with a post-hoc adjustment (see figure legends) when comparing data with multiple samples.

### Phylogenetic analyses

A protein sequence database was prepared comprising the annotated sequences from the 617 publicly available complete *Vibrio* genome assemblies. An initial set of potential homologous protein sequence pairs occurring within the same operon were identified by BLASTP [131] (version ncbi-blast-2.13.0+). Multiple sequence alignments (MSA) for these sequences were generated using MUSCLE [132] (version 3.8.31, parameters: -diags -sv -distance1 kbit20_3). Profile HMMs constructed from each set of MSA were searched against the complete *Vibrio* protein sequence database using HMMER [133] version 3.2.1. The resulting hits were iteratively used to build further profile HMMs while newer hits were being found from the previous iteration. Potential RcbA and RcbB homolog pairs were required to co-occur on the same chromosome or contig sequence and on the same strand, potentially from the same operon. TPR counts per sequence were estimated using TPRpred [134] (with HMM tpr2.8.pp). Single copy core genes were identified from each of the *Vibrio* assemblies using BUSCO 5.7.1 searching against the vibrionales_odb10 database [135]. MSA was performed on the protein sequences for each of the 133 common core genes from all 617 assemblies using MUSCLE. The individual amino acid residues were substituted with the corresponding codon sequences to obtain the MSA of the coding sequences, while maintaining information on any changes in degenerate positions to preserve codon integrity and the amino acid context. The phylogenetic tree was calculated as the best scoring Maximum Likelihood bootstrap tree using RAxML [136] version 8.2.12 (parameters: -m GTRGAMMA -B 0.03 -# 100). The tree was visualized and exported using the Integrated Tree of Life [137] web service available at https://itol.embl.de.

## Supporting information

S1 Table Strains and plasmids. (DOCX)

S2 Table *rcbAB* homologs in *Vibrio* genomes. (XLSX)

S3 Table *rcbAB* homologs in non-*Vibrio* genomes. (XLSX)

S1 Fig The *rcbAB* operon regulates calcium-induced *cab* expression. Plot of wildtype *V. vulnificus* (WT) and ∆*rcbAB* mutant (Tn) strains bearing a *P*_*cabA*_*lacZ* reporter grown in LB without or supplemented with 15 mM CaCl_2_ (LB + Ca^2+^). Bars indicate the respective mean values and error bars represent the standard deviation of triplicate assays. Statistically significant differences between samples (****p < 0.0001; ns, no significant difference) were determined by unpaired student’s t-test (two-tailed).
(TIFF)

S2 Fig CaCl_2_ inhibits *V. vulnificus* swimming motility independent of *rcbAB.* Bar plot of the motility zones for wildtype *V. vulnificus* and the *rcbA*, *rcbB* and *rcbAB* deletion mutants (∆*rcbA,* ∆*rcbB* and ∆*rcbAB*, respectively) in soft agar lacking (orange) or supplemented with the indicated concentration of CaCl_2_. Bars show the respective mean values for each strain and error bars represent the standard deviation of triplicate assays. No statistically significant difference (ns) relative to WT was found among strains at the same CaCl_2_ concentration (one-way ANOVA with a Dunnett’s multiple comparisons post-hoc test).
(TIFF)

S3 Fig RcbA and RcbB co-operatively decrease motility independent of CaCl_2_ concentration. Plot of motility by wildtype *V. vulnificus* and the *rcbAB* deletion mutant (∆*rcbAB*) carrying the empty expression plasmid or expressing (+) *rcbA*, *rcbB* or *rcbAB* grown in soft agar lacking (orange) or supplemented with the indicated concentration of CaCl_2_. Bars show the respective mean values for each strain and error bars represent the standard deviation of triplicate assays. Statistically significant differences relative to wildtype were determined by one-way ANOVA with a Dunnett’s multiple comparisons post-hoc test (**p < 0.01; ns, no significant difference). Expression was induced with IPTG (10 µM).
(TIFF)

S4 Fig RcbA and RcbB co-operatively increase cellular c-di-GMP levels independent of CaCl_2_ concentration. Plot of intracellular c-di-GMP levels in wildtype *V. vulnificus* and the *rcbAB* deletion mutant (∆*rcbAB*) that carries the empty expression plasmid or express (+) *rcbA*, *rcbB* or *rcbAB* grown in LB lacking (orange) or supplemented with the indicated concentration of CaCl_2_. Bars show the respective mean values for each strain and error bars represent the standard deviation of triplicate assays. Statistically significant differences relative to wildtype were determined by one-way ANOVA with a Dunnett’s multiple comparisons post-hoc test (***p < 0.001; ns, no significant difference). Expression was induced with IPTG (10 µM).
(TIFF)

S5 Fig RcbB activity is dependent on a functional GGDEF domain. A, plot of biofilm formation (OD_595_/OD_600_) by ∆*rcbB* cells harboring the empty expression plasmid (control) or plasmids expressing (+) *rcbB* or a catalytic mutant bearing the A-to-E mutation, GGAEF). B, a plot of swimming motility zones in IO20 soft agar by the same strains in A. Bars indicate the respective mean values and error bars represent the standard deviation of triplicate assays. Statistically significant differences (****p < 0.0001; ns, no significant difference) relative to the control were determined by one-way ANOVA with a Dunnett’s multiple comparisons post-hoc test. Expression was induced with 1 µM IPTG in A and 10 µM IPTG in B.
(TIFF)

S6 Fig Expression of *rcbAB* slows motility of *E. coli* cells. Plot of swimming motility zones in soft agar for several *E. coli* strains (A) and for MG1655 cells (B) carrying the empty expression vector (Con) or the plasmid with *rcbA*, *rcbB* or *rcbAB* (without or with (+) 10 µM IPTG; separated by the vertical dotted line). Bars indicate the respective mean values and error bars represent the standard deviation of triplicate assays. Statistically significant differences (*p < 0.05, ****p < 0.0001; ns, no significant difference) relative to the control were determined by one-way ANOVA with a Dunnett’s multiple comparisons post-hoc test.
(TIFF)

S7 Fig Production of RcbA, RcbB and variants lacking the TPR domain in *V. vulnificus.* A, western blot of ∆*rcbA* extracts containing HIS-tagged RcbA or ∆*rcbB* extracts containing HA-tagged RcbB. Expression was induced with the IPTG concentrations indicated below each lane and the proteins were detected with anti-HIS and anti-HA antibodies, respectively. B, alignments of AlphaFold 3 predicted monomer structures of RcbA (purple) with RcbA^∆TPR^ (pink), and RcbB (green) with RcbB^∆TPR^ (blue). C, western blot of ∆*rcbA* extracts containing HIS-tagged RcbA^∆TPR^ or HA-tagged RcbB^∆TPR^. Expression and detection conditions were as in A.
(TIFF)

S8 Fig Fluorescently tagged RcbA and RcbB proteins function to promote biofilm formation. Biofilm formation (OD_595_/OD_600_) by ∆*rcbAB* cells harboring empty plasmids (control) or expressing (+) *rcbA-mRuby3* (RcbAmRu), *rcbB-mNeonGreen* (RcbBmNG) or *rcbA-mRuby3-rcbB-mNeonGreen* (RcbAmRu/RcbBmNG). Bars indicate the respective mean values and error bars represent the standard deviation of triplicate assays. Statistically significant differences (****p < 0.0001; ns, no significant difference) relative to control cells were determined by one-way ANOVA with a Dunnett’s multiple comparisons post-hoc test. Expression was induced with 1 µM IPTG.
(TIFF)

S9 Fig Flagellar synthesis is dependent on *flrA* but not *hubP.* Representative fluorescence images of NanoOrange stained ∆*hubP* and ∆*flrA* cells. White arrows denote flagella. Scale bar = 1 µm.
(TIFF)

S10 Fig RcbA anchors RcbB to the pole in the absence of flagella. From left to right, representative phase, fluorescence and overlay images of RcbA-mRuby3 (A), RcbB-mNeonGreen (B), or both (C) in ∆*flrA*∆*rcbAB* cells. Scale bar = 1 μm. Expression was induced with 10 µM IPTG.
(TIFF)

S11 Fig Proposed structural transition of RcbB upon association with RcbA. AlphaFold 3 predicted monomer structures of RcbB alone (blue) and complexed (green) with RcbA. A GTP molecule (stick configuration) is modeled bound at the active site. The grey arrow denotes rotation of the DGC domain relative to the aligned TPR domains.
(TIFF)
