# Supplementary material for: Spatio-genetically coordinated TPR domain-containing proteins modulate c-di-GMP signaling in Vibrio vulnificus
Source: PLoS Pathog. 2025 Jul 16;21(7):e1013353. doi: 10.1371/journal.ppat.1013353 (PMC12282931; doi:10.1371/journal.ppat.1013353)
Supplement: S1 Table — (DOCX) [file ppat.1013353.s001.docx]

**Table S1. Strains and plasmids used in this study.**

| **Strain** | **Description** | **Source or reference** |
| --- | --- | --- |
| ***E. coli*** | | |
| S17.1λ*pir* | *TpR SmR recA, thi, pro, hsdR-M+RP4:2-Tc:Mu: Km Tn7 λpir* | (1) |
| GM48 | *F- thr leu thi lacY galK galT ara fhuA tsx dam dcm glnV44* | (2) |
| TOP10 | *F- mcrA Δ(mrr-hsdRMS-mcrBC) φ80lacZΔM15 ΔlacΧ74 recA1 araD139 Δ(ara-leu) 7697*  *galU galK rpsL (Str^R^) endA1 nupG λ-* | (2) |
| EPI100 | *F- mcrA Δ(mrr-hsdRMS-mcrBC) ϕ80dlacZΔM15 ΔlacX74 recA1 endA1 araD139 Δ(ara, leu) 7697*  *galU galK rpsL (Str^R^) nupG λ-* | (2) |
| DH5α | *F^–^ φ80lacZΔM15 Δ(lacZYA-argF) U169 recA1 endA1 hsdR17 (r_K_^–^, m_K_^+^) phoA supE44 λ^–^thi-1 gyrA96 relA1* | (3) |
| BTH101 | BACTH strain *F-, cya-99, araD139, galK16, rpsL1 (Str^R^), hsdR2, mcrA1, mcrB1* | (4) |
|  | | |
| ***V. vulnificus*** | | |
| ATCC27562 | Rif^R^ strain | (5) |
| Δ*rcbA::Tpk7* | *rcbA* deletion mutant, Tp^R^ | This work |
| Δ*rcbB::Tpk7* | *rcbB* deletion mutant, Tp^R^ | This work |
| Δ*rcbAB::Tpk7* | *rcbAB* deletion mutant, Tp^R^ | This work |
| Δ*hubP::Cmk7* | *hubP* deletion mutant, Tp^R^ | This work |
| Δ*flrA::Cmk7* | *flrA* deletion mutant, Tp^R^ | This work |
| Δ*rcbAB::Tpk7* Δ*hubP::Cmk7* | *rcbAB* and *hubP* deletion mutant, Tp^R^ and Cm^R^ | This work |
| Δ*rcbAB::Tpk7* Δ*flrA::Cmk7* | *rcbAB* and *flrA* deletion mutant, Tp^R^ and Cm^R^ | This work |
| Δ*ebgA::P_brpA_-lacZ^Ec^-Cmk7* | *P_brpA_-lacZ^Ec^* reporter strain, Cm^R^ | This work |
| Δ*ebgA::P_cabA_-lacZ^Ec^-Cmk7* | *P_cabA_-lacZ^Ec^* reporter strain, Cm^R^ | This work |
|  | |  |
| **Plasmid** | |  |
| pSU38GT | *p15A ori rep*, *P_bad_* arabinose-inducible promoter, Gm^R^ | (6) |
| pSU38GT-*fliM-bfp* | Expression of fluorescently tagged *fliM* | This work |
| pC2X6HIST | *pMB1 ori rep*, *P_tac_* IPTG-inducible promoter, Ap^R^ | (7) |
| pC2X6HIST-*rcbA* | Expression of *rcbA* | This work |
| pC2X6HIST-*rcbB* | Expression of *rcbB* | This work |
| pC2X6HIST-*rcbAB* | Expression of *rcbAB* | This work |
| pC2X6HIST-*rcbA*^Δ^*^tpr^* | Expression of *rcbA* variant lacking the TPR domain | This work |
| pC2X6HIST-*rcbB*^Δ^*^tpr^* | Expression of *rcbB* variant lacking the TPR domain | This work |
| pC2X6HIST-*rcbA*^Δ^*^tpr^-rcbB* | Expression of *rcbA* variant lacking the TPR domain and *rcbB* | This work |
| pC2X6HIST-*rcbA*^Δ^*^C^-rcbB* | Expression of *rcbA* variant lacking the C-terminal region and *rcbB* | This work |
| pC2X6HIST-*rcbA-mRuby3* | Expression of fluorescently tagged *rcbA* | This work |
| pC2X6HIST-*rcbB-mNeonGreen* | Expression of fluorescently tagged *rcbB* | This work |
| pC2X6HIST-*rcbA-mRuby3-rcbB-mNeonGreen* | Expression of fluorescently tagged *rcbA* and *rcbB* | This work |
| pC2X6HIST-*rcbB*^Δ^*^tpr^-mNeonGreen* | Expression of fluorescently tagged *rcbA* variant lacking the TPR domain | This work |
| pC2X6HIST-*rcbA^tpr6/7^-mRuby3-rcbB^tpr6/7^-mNeonGreen* | Expression of fluorescently tagged variants of *rcbA* and *rcbB* lacking TPR1-5 | This work |
| pC2X6HIST-*rcbA^tpr7^-mRuby3-rcbB^tpr7^-mNeonGreen* | Expression of fluorescently tagged variants of *rcbA* and *rcbB* lacking TPR1-6 | This work |
| pC2X6HIST-*rcbA* | Expression of HIS-tagged *rcbA* | This work |
| pC2X6HAT-*rcbB* | Expression of HA-tagged *rcbB* | This work |
| pC2X6HIST-*rcbA*^Δ^*^TPR^* | Expression of HIS-tagged *rcbA* variant lacking the TPR domain | This work |
| pC2X6HA-*rcbB*^Δ^*^TPR^* | Expression of HA-tagged *rcbB* variant lacking the TPR domain | This work |
| pKNT25 | BACTH vector, Kn^R^ | (4) |
| pUT18 | BACTH vector, Ap^R^ | (4) |
| pUT18-*zip* | BACTH positive control vector expressing *zip* | (4) |
| pKT25-*zip* | BACTH positive control vector expressing *zip* | (4) |
| pUT18-*rcbA* | BACTH vector expressing *rcbA* | This work |
| pKNT25-*rcbA* | BACTH vector expressing *rcbA* | This work |
| pUT18-*rcbB* | BACTH vector expressing *rcbB* | This work |
| pKNT25-*rcbB* | BACTH vector expressing *rcbB* | This work |
| pUT18-*rcbA^TPR^* | BACTH vector expressing *rcbA* variant lacking the TPR domain | This work |
| pKNT25-*rcbA^TPR^* | BACTH vector expressing *rcbA* variant lacking the TPR domain | This work |
| pUT18-*rcbB^TPR^* | BACTH vector expressing *rcbB* variant lacking the TPR domain | This work |
| pKNT25-*rcbB^TPR^* | BACTH vector expressing *rcbB* variant lacking the TPR domain | This work |

**References**

1. Simon R, Priefer U, Puhler A. A broad host range mobilization system for in vivo genetic engineering: transposon mutagenesis in Gram negative bacteria. Bio/Technology. 1983;1:784–91.

2. Jeong H, Barbe V, Lee CH, Vallenet D, Yu DS, Choi SH, et al. Genome Sequences of Escherichia coli B strains REL606 and BL21(DE3). J Mol Biol. 2009;394(4):644–52.

3. Taylor RG, Walker DC, Mclnnes RR. E.coli host strains significantly affect the quality of small scale plasmid DNA preparations used for sequencing. Nucleic Acids Res. 1993;21(7):1677–8.

4. Karimova G, Ullmann A, Ladant D. A bacterial two-hybrid system that exploits a cAMP signaling cascade in Escherichia coli. Methods Enzym. 2000;328:59–73.

5. Nakhamchik A, Wilde C, Rowe-Magnus DA. Identification of a Wzy polymerase required for group IV capsular polysaccharide and lipopolysaccharide biosynthesis in Vibrio vulnificus. Infect Immun [Internet]. 2007;75(12):5550–8. Available from: <http://iai.asm.org/cgi/content/abstract/75/12/5550>

6. Guo Y, Rowe-Magnus DA. Overlapping and unique contributions of two conserved polysaccharide loci in governing distinct survival phenotypes in Vibrio vulnificus. Environ Microbiol [Internet]. 2011;13(11):2888–990. Available from: <http://doi.wiley.com/10.1111/j.1462-2920.2011.02564.x>

7. Chodur DM, Guo L, Pu M, Bruger E, Fernandez N, Waters C, et al. The Proline Variant of the W[F/L/M][T/S]R Cyclic Di-GMP Binding Motif Suppresses Dependence on Signal Association for Regulator Function. J Bacteriol [Internet]. 2017;199(19):e00344-17. Available from: <https://jb.asm.org/content/199/19/e00344-17.abstract>
