# Supplementary figures and images for: Spatio-genetically coordinated TPR domain-containing proteins modulate c-di-GMP signaling in Vibrio vulnificus
Source: PLoS Pathog. 2025 Jul 16;21(7):e1013353. doi: 10.1371/journal.ppat.1013353 (PMC12282931; doi:10.1371/journal.ppat.1013353)

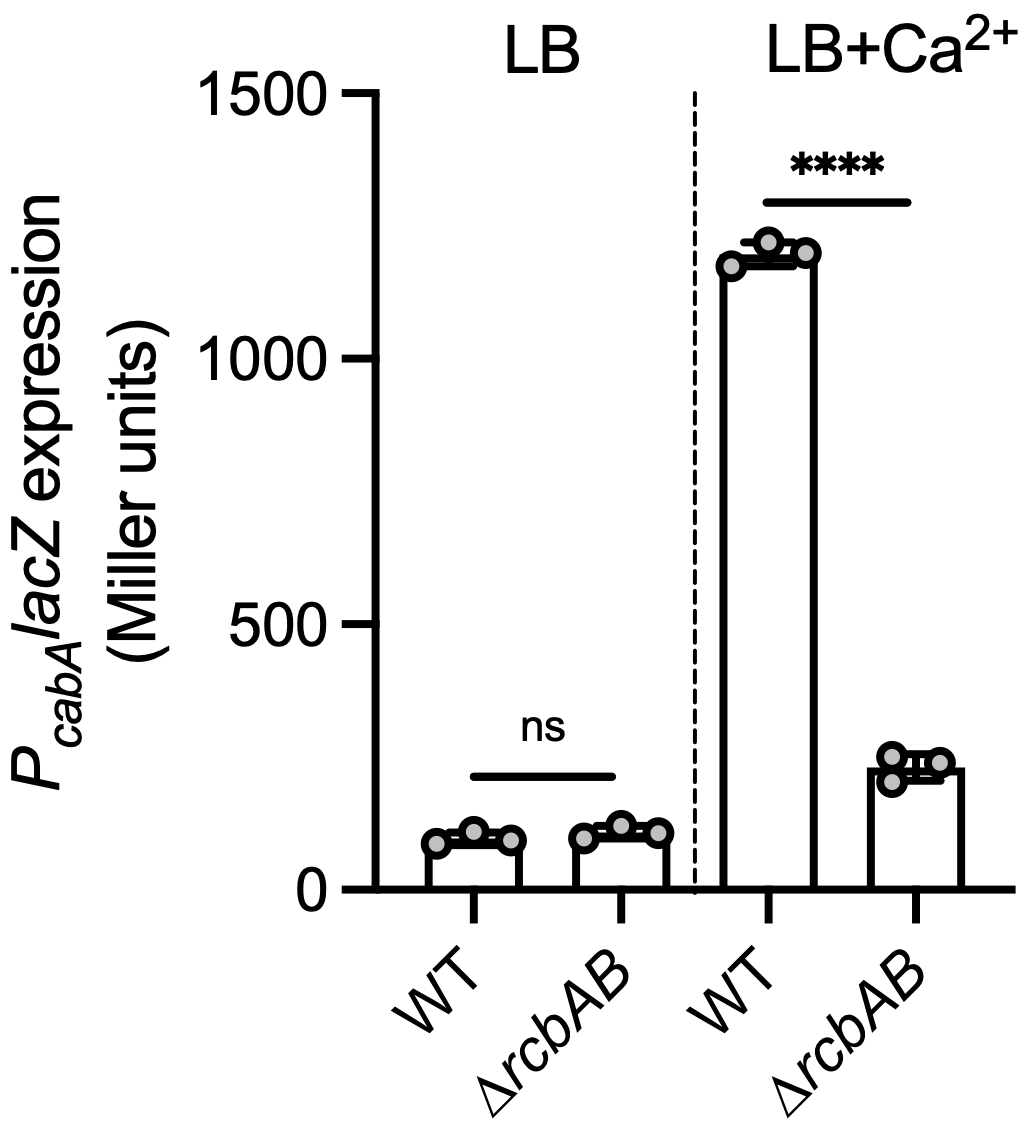

Supplement: S1 Fig — Plot of wildtype V. vulnificus (WT) and ∆rcbAB mutant (Tn) strains bearing a PcabAlacZ reporter grown in LB without or supplemented with 15 mM CaCl2 (LB + Ca2+). Bars indicate the respective mean values and error bars represent the standard deviation of triplicate assays. Statistically significant differences between samples (****p < 0.0001; ns, no significant difference) were determined by unpaired student’s t-test (two-tailed). (TIFF) [file ppat.1013353.s004.tiff]

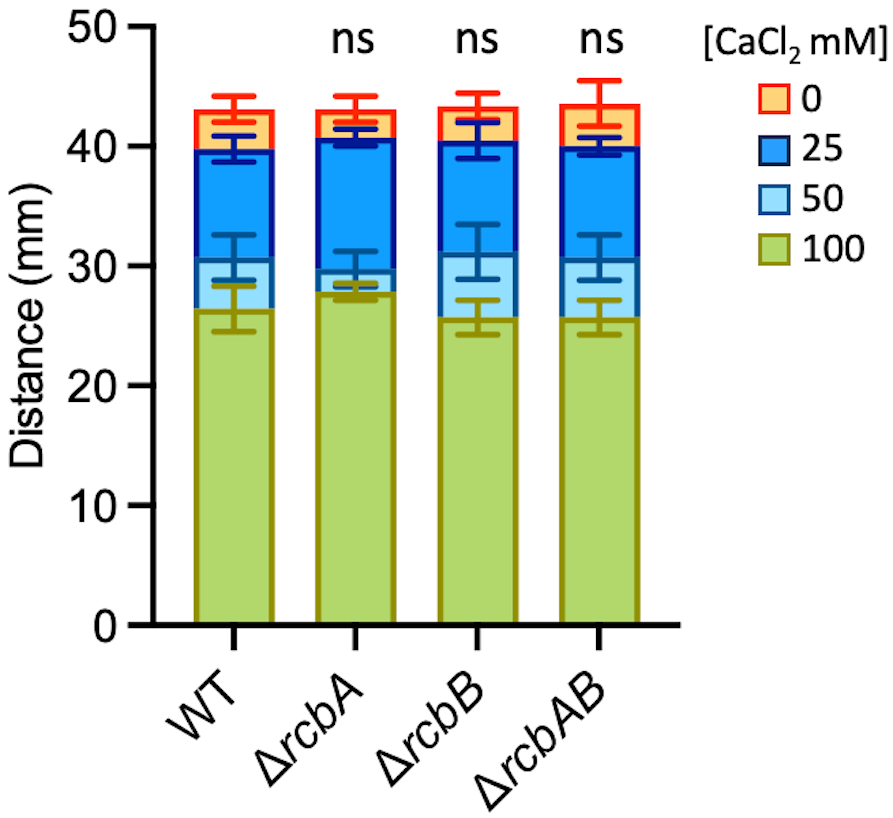

Supplement: S2 Fig — Bar plot of the motility zones for wildtype V. vulnificus and the rcbA, rcbB and rcbAB deletion mutants (∆rcbA, ∆rcbB and ∆rcbAB, respectively) in soft agar lacking (orange) or supplemented with the indicated concentration of CaCl2. Bars show the respective mean values for each strain and error bars represent the standard deviation of triplicate assays. No statistically significant difference (ns) relative to WT was found among strains at the same CaCl2 concentration (one-way ANOVA with a Dunnett’s multiple comparisons post-hoc test). (TIFF) [file ppat.1013353.s005.tiff]

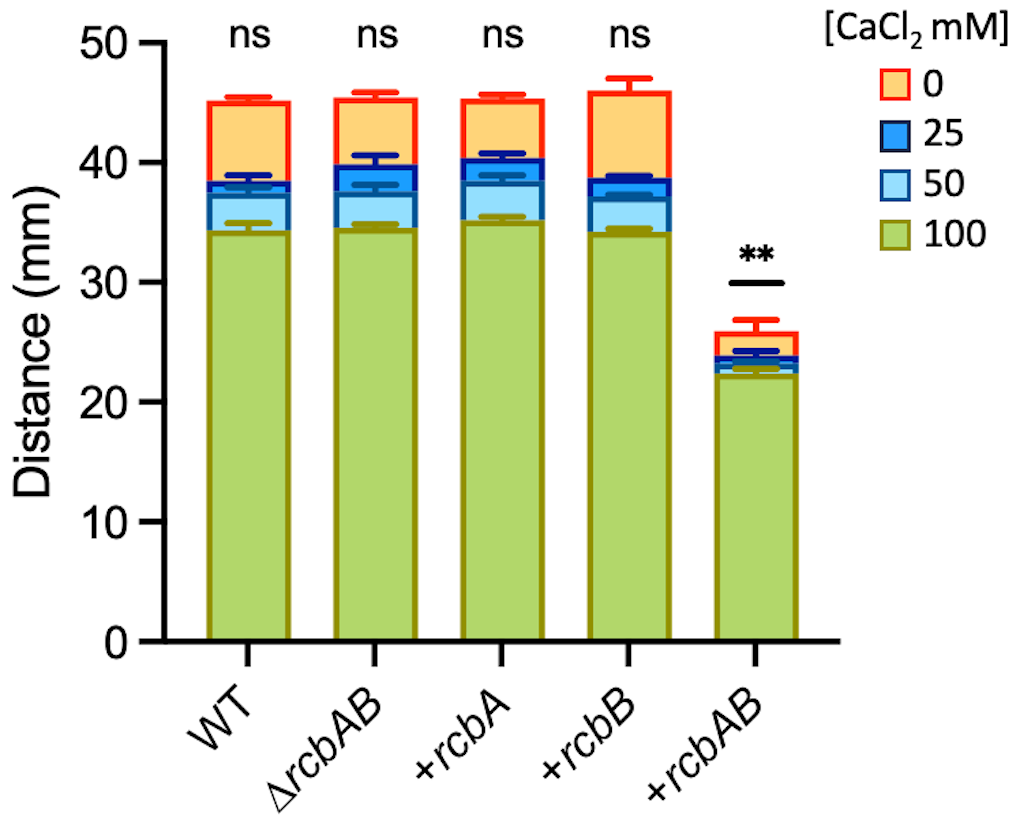

Supplement: S3 Fig — Plot of motility by wildtype V. vulnificus and the rcbAB deletion mutant (∆rcbAB) carrying the empty expression plasmid or expressing (+) rcbA, rcbB or rcbAB grown in soft agar lacking (orange) or supplemented with the indicated concentration of CaCl2. Bars show the respective mean values for each strain and error bars represent the standard deviation of triplicate assays. Statistically significant differences relative to wildtype were determined by one-way ANOVA with a Dunnett’s multiple comparisons post-hoc test (**p < 0.01; ns, no significant difference). Expression was induced with IPTG (10 µM). (TIFF) [file ppat.1013353.s006.tiff]

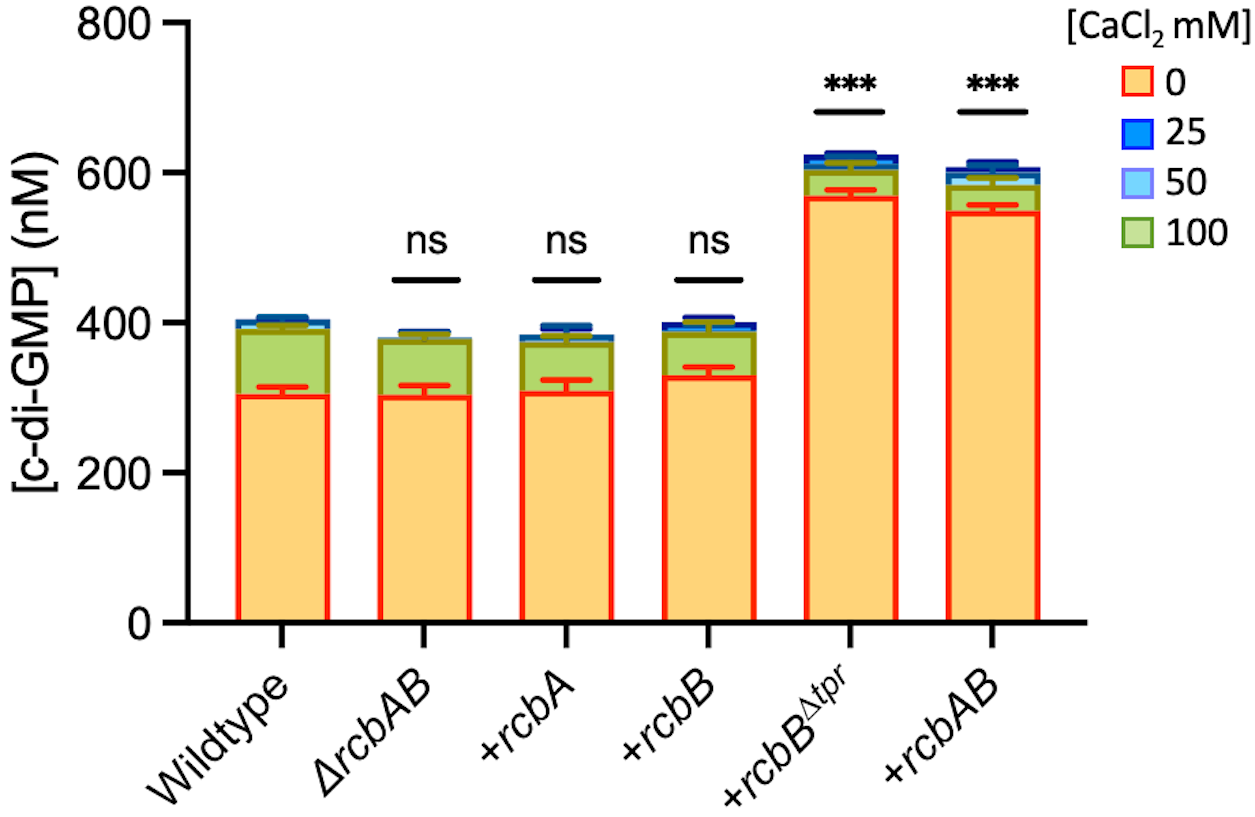

Supplement: S4 Fig — Plot of intracellular c-di-GMP levels in wildtype V. vulnificus and the rcbAB deletion mutant (∆rcbAB) that carries the empty expression plasmid or express (+) rcbA, rcbB or rcbAB grown in LB lacking (orange) or supplemented with the indicated concentration of CaCl2. Bars show the respective mean values for each strain and error bars represent the standard deviation of triplicate assays. Statistically significant differences relative to wildtype were determined by one-way ANOVA with a Dunnett’s multiple comparisons post-hoc test (***p < 0.001; ns, no significant difference). Expression was induced with IPTG (10 µM). (TIFF) [file ppat.1013353.s007.tiff]

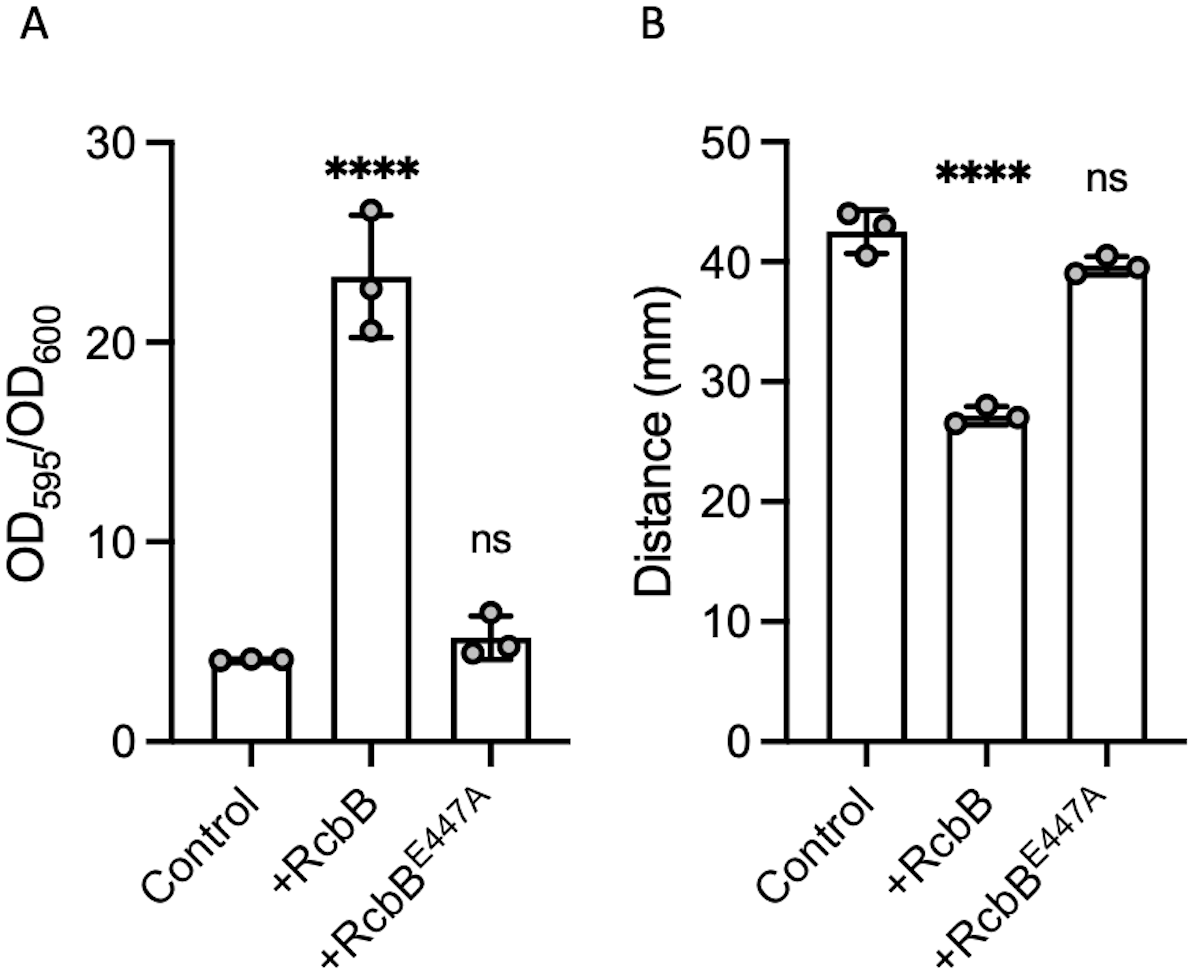

Supplement: S5 Fig — A, plot of biofilm formation (OD595/OD600) by ∆rcbB cells harboring the empty expression plasmid (control) or plasmids expressing (+) rcbB or a catalytic mutant bearing the A-to-E mutation, GGAEF). B, a plot of swimming motility zones in IO20 soft agar by the same strains in A. Bars indicate the respective mean values and error bars represent the standard deviation of triplicate assays. Statistically significant differences (****p < 0.0001; ns, no significant difference) relative to the control were determined by one-way ANOVA with a Dunnett’s multiple comparisons post-hoc test. Expression was induced with 1 µM IPTG in A and 10 µM IPTG in B. (TIFF) [file ppat.1013353.s008.tiff]

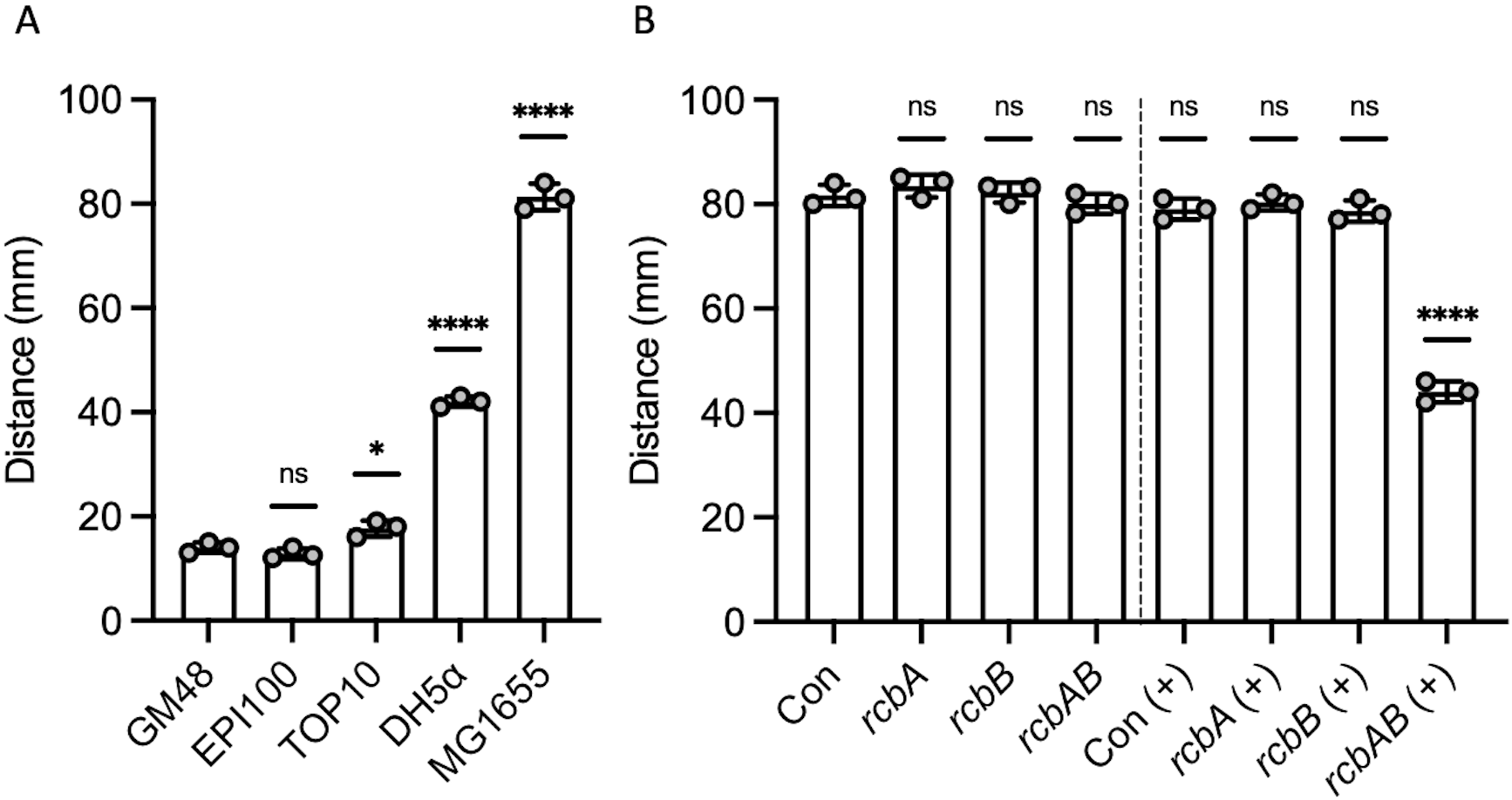

Supplement: S6 Fig — Plot of swimming motility zones in soft agar for several E. coli strains (A) and for MG1655 cells (B) carrying the empty expression vector (Con) or the plasmid with rcbA, rcbB or rcbAB (without or with (+) 10 µM IPTG; separated by the vertical dotted line). Bars indicate the respective mean values and error bars represent the standard deviation of triplicate assays. Statistically significant differences (*p < 0.05, ****p < 0.0001; ns, no significant difference) relative to the control were determined by one-way ANOVA with a Dunnett’s multiple comparisons post-hoc test. (TIFF) [file ppat.1013353.s009.tiff]

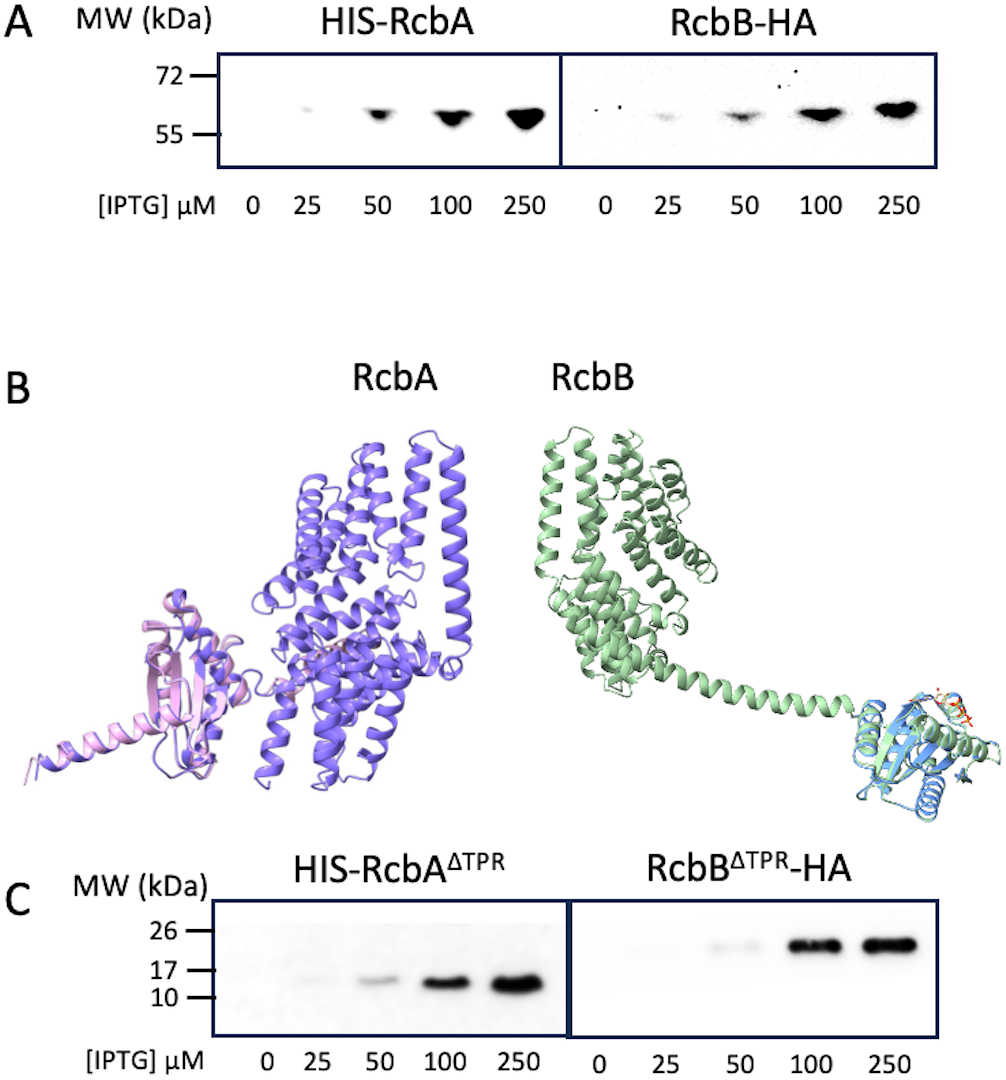

Supplement: S7 Fig — A, western blot of ∆rcbA extracts containing HIS-tagged RcbA or ∆rcbB extracts containing HA-tagged RcbB. Expression was induced with the IPTG concentrations indicated below each lane and the proteins were detected with anti-HIS and anti-HA antibodies, respectively. B, alignments of AlphaFold 3 predicted monomer structures of RcbA (purple) with RcbA∆TPR (pink), and RcbB (green) with RcbB∆TPR (blue). C, western blot of ∆rcbA extracts containing HIS-tagged RcbA∆TPR or HA-tagged RcbB∆TPR. Expression and detection conditions were as in A. (TIFF) [file ppat.1013353.s010.tiff]

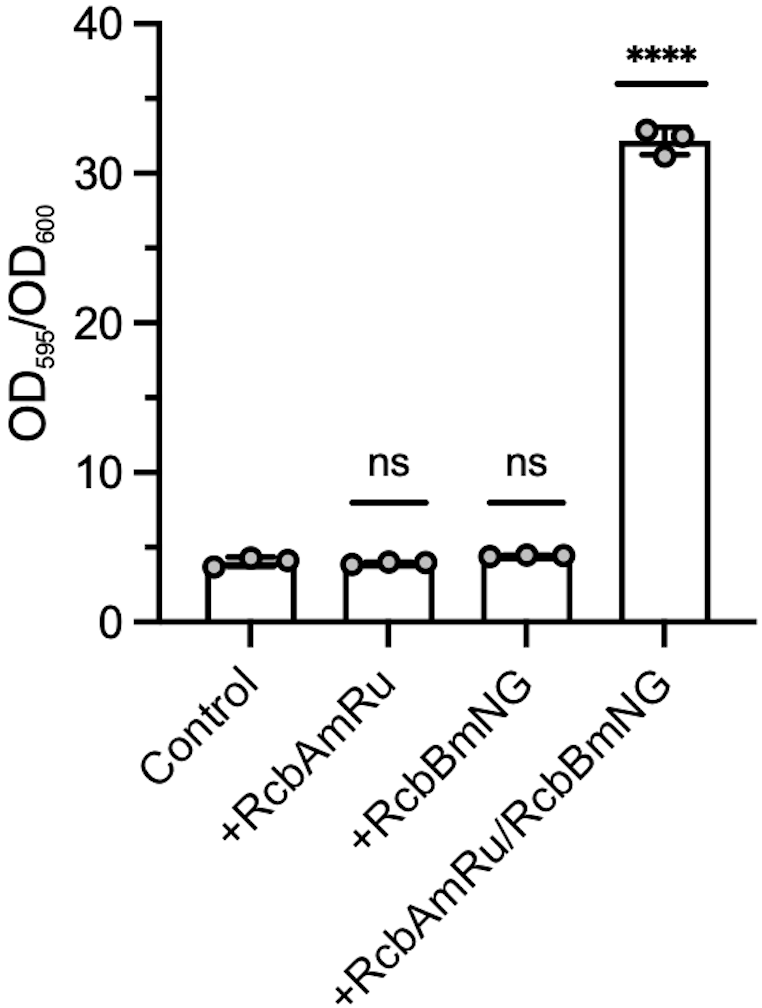

Supplement: S8 Fig — Biofilm formation (OD595/OD600) by ∆rcbAB cells harboring empty plasmids (control) or expressing (+) rcbA-mRuby3 (RcbAmRu), rcbB-mNeonGreen (RcbBmNG) or rcbA-mRuby3-rcbB-mNeonGreen (RcbAmRu/RcbBmNG). Bars indicate the respective mean values and error bars represent the standard deviation of triplicate assays. Statistically significant differences (****p < 0.0001; ns, no significant difference) relative to control cells were determined by one-way ANOVA with a Dunnett’s multiple comparisons post-hoc test. Expression was induced with 1 µM IPTG. (TIFF) [file ppat.1013353.s011.tiff]

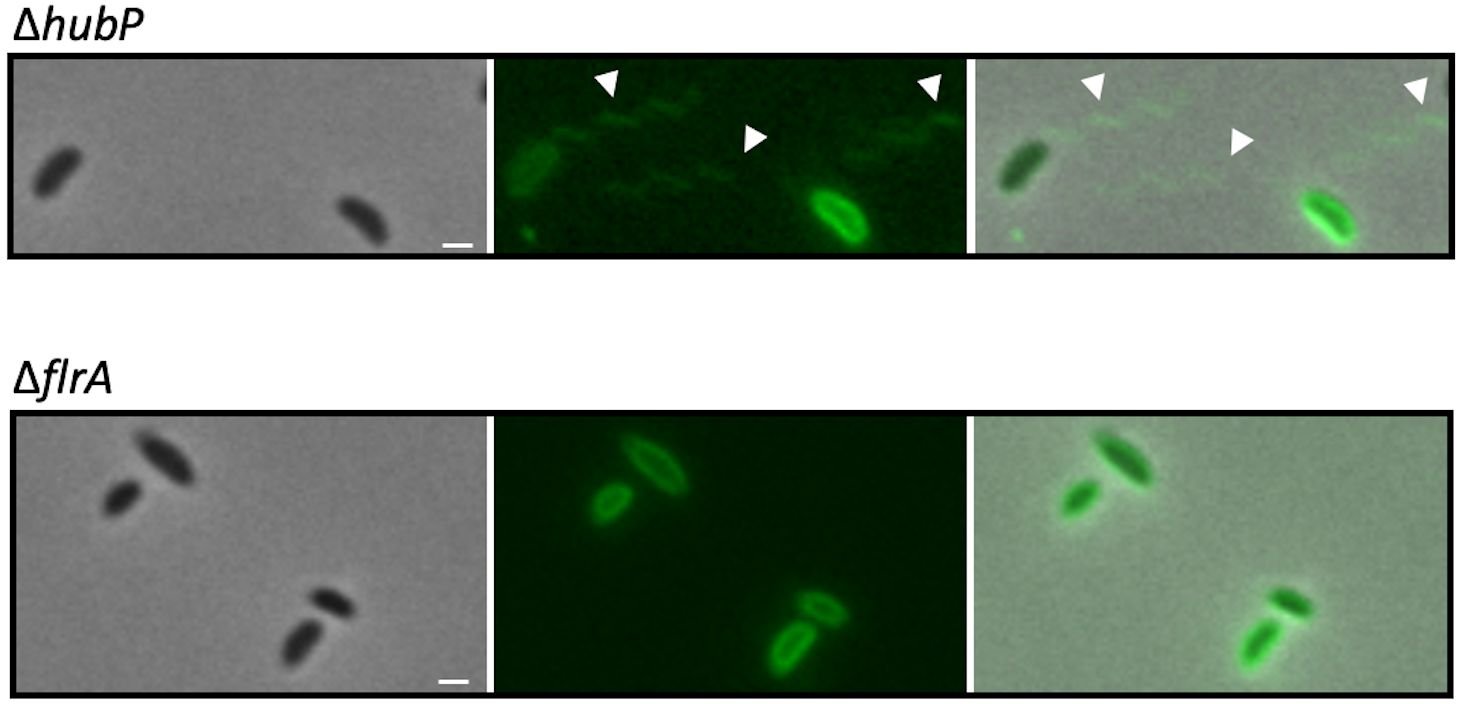

Supplement: S9 Fig — Representative fluorescence images of NanoOrange stained ∆hubP and ∆flrA cells. White arrows denote flagella. Scale bar = 1 µm. (TIFF) [file ppat.1013353.s012.tiff]

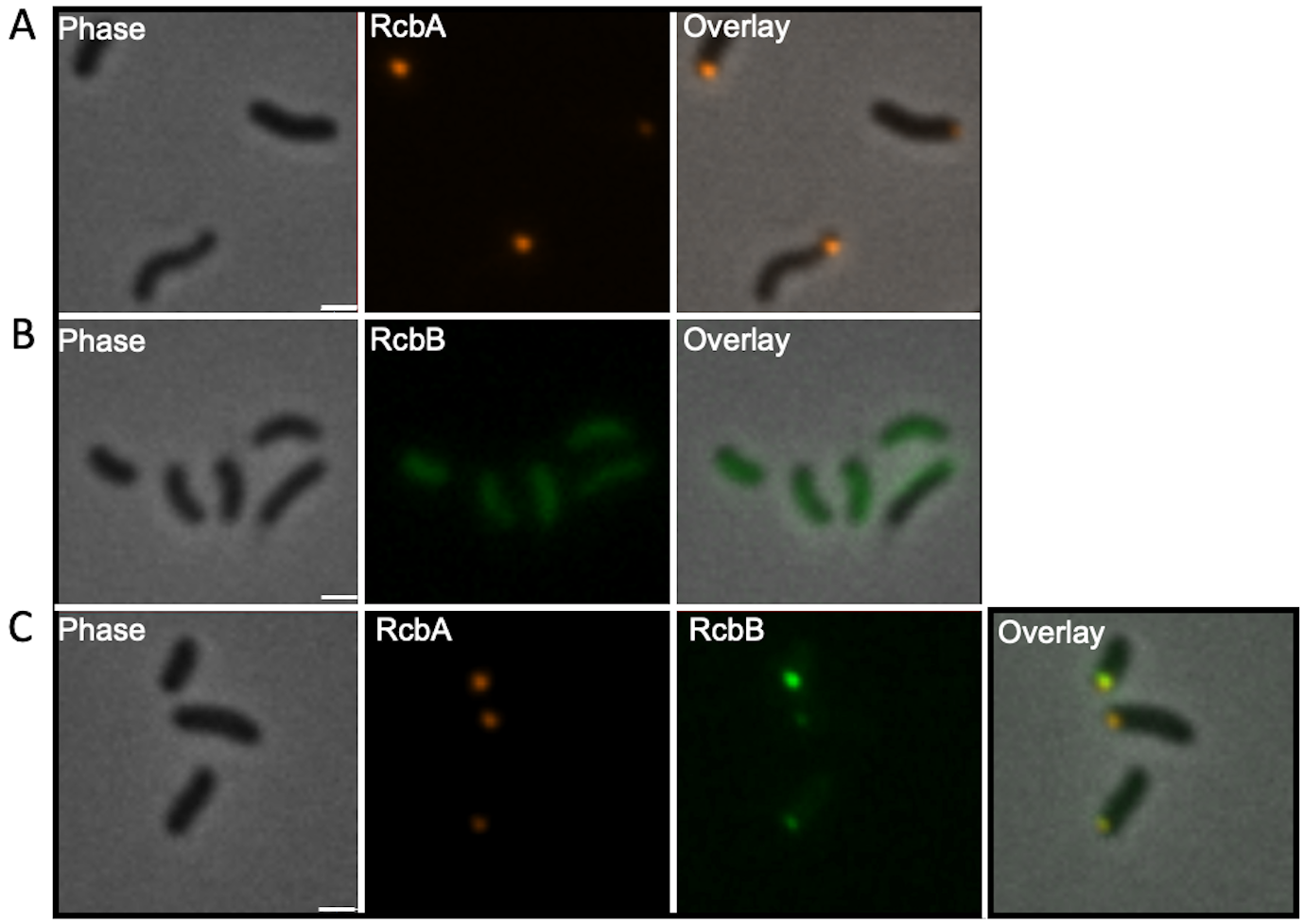

Supplement: S10 Fig — From left to right, representative phase, fluorescence and overlay images of RcbA-mRuby3 (A), RcbB-mNeonGreen (B), or both (C) in ∆flrA∆rcbAB cells. Scale bar = 1 μm. Expression was induced with 10 µM IPTG. (TIFF) [file ppat.1013353.s013.tiff]

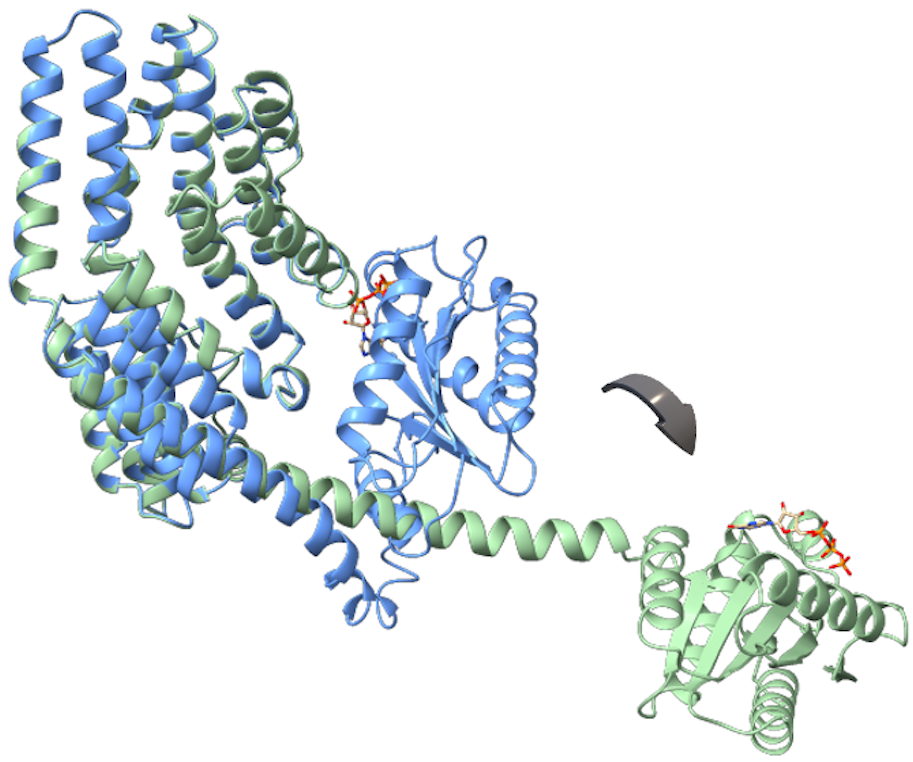

Supplement: S11 Fig — AlphaFold 3 predicted monomer structures of RcbB alone (blue) and complexed (green) with RcbA. A GTP molecule (stick configuration) is modeled bound at the active site. The grey arrow denotes rotation of the DGC domain relative to the aligned TPR domains. (TIFF) [file ppat.1013353.s014.tiff]
